# New insights on repellent recognition by *Anopheles gambiae* odorant-binding protein 1

**DOI:** 10.1371/journal.pone.0194724

**Published:** 2018-04-03

**Authors:** George Tzotzos, Jim N. Iley, Elaine A. Moore

**Affiliations:** 1 Ferrogasse, Vienna, Austria; 2 School of Life, Health and Chemical Sciences, Open University, Milton Keynes, United Kingdom; USDA Agricultural Research Service, UNITED STATES

## Abstract

It is generally recognized that insect odorant binding proteins (OBPs) mediate the solubilisation and transport of hydrophobic odorant molecules and contribute to the sensitivity of the insect olfactory system. However, the exact mechanism by which OBPs deliver odorants to olfactory receptors and their role, if any, as selectivity filters for specific odorants, are still a matter of debate. In the case of *Anopheles gambiae*, recent studies indicate that ligand discrimination is effected through the formation of heterodimers such as AgamOBP1 and AgamOBP4 (odorant binding proteins 1 and 4 from *Anopheles gambiae*). Furthermore, AgamOBPs have been reported to be promiscuous in binding more than one ligand simultaneously and repellents such as DEET *(N*,*N-diethyl-3-toluamide)* and 6-MH *(6-methyl-5-hepten-2-one)* interact directly with mosquito OBPs and/or compete for the binding of attractive odorants thus disrupting OBP heterodimerisation. In this paper, we propose mechanisms of action of DEET and 6-MH. We also predict that ligand binding can occur in several locations of AgamOBP1 with partial occupancies and propose structural features appropriate for repellent pharmacophores.

## Introduction

Mosquitoes occupy the leading place among insect vectors responsible for the transmission of parasitic and viral infections, such as malaria, yellow fever, dengue and Chikungunya. The World Health Organisation estimated that the number of deaths caused by malaria in 2012 was well in excess of 600 000 representing approx. 17% of the global burden of all infectious diseases [1]. To date, preventive and/or therapeutic interventions against malaria have been frustrated either by the limited efficacy of malaria vaccines [2] or the emergence of resistance to modern drugs such as artemisin [3]. In view of these problems, alternative biotechnological approaches aimed at reducing contact between mosquitoes and human hosts are being considered. One such approach entails the disruption of the olfactory behaviour of the insect vectors.

**Fig 1:**
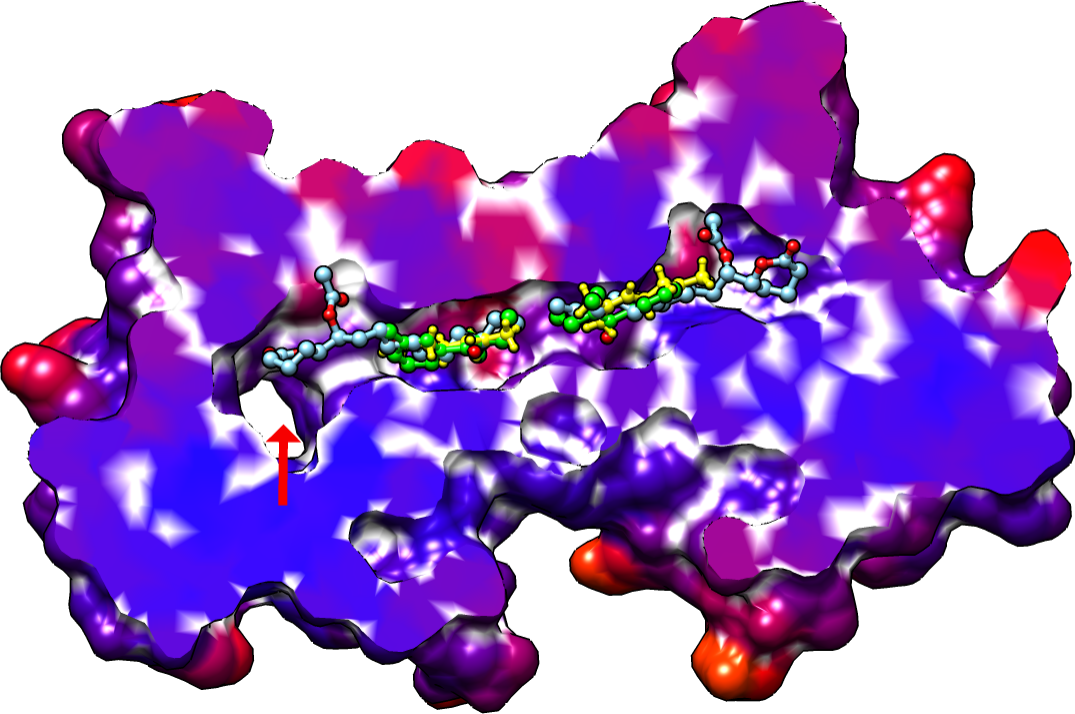
Superimposed structures of CquiOBP1 in complex with MOP (PDB ID: 3OGN) (cyan) and AgamOBP1 in complex with DEET (PDB ID: 3N7H) (green) and 6-MH (PDB ID: 4FQT) (yellow). RMSD between 125 atom pairs of 3N7H and 4FQT is 0.034 nm. RMSD between 124 atom pairs of 3N7H and 3OGN is 0.037 Å. Serendipitous ligands not shown. The side pocket opening to bulk solvent is shown by the arrow at the l.h.s of the image.

The insect olfactory system is extremely complex involving hundreds of diverse transmembrane odorant receptor proteins (ORs) located on olfactory receptor neurons (ORNs) that are found in sensilla of the antennae, maxillary palps and the proboscis. The biochemical and structural properties of ORs are reviewed extensively elsewhere [4] [5]. Interactions between odorant molecules and ORs are translated into ion gradient potential signals by transductory proteins and/or ligand-gated ion channels, which are recognised by the central nervous system. Targeting ORs to render mosquitoes “odour blind” has proven elusive [6]. Attention has been turned instead to odorant binding proteins (OBPs), which constitute another component of the insect olfactory system. These proteins, found in the sensillum lymph of the antennae, are thought to facilitate the solubilisation and transport of odorant molecules [7] [8]. They may also protect odorants from degradation by odorant degrading enzymes of the lymph and/or “scavenge” excess ligand to avoid secondary stimulation of the neurones [9] [10]. Although, OBPs have also been shown to enhance olfactory sensitivity when co-expressed with ORs [11] [12], it is still debatable whether they play a role in odorant selectivity and/or OR activation [5]. The great diversity of OBPs (> 50 in the case of most mosquito species) and their low sequence identity suggest that these proteins may serve as selectivity filters, binding odorants of different chemical classes with different affinities. With regard to their role in OR activation, experimental evidence points to two different models. According to the first one, receptor response is elicited by the OBP-ligand complex [13] [14], whereas the second one suggests that receptors are activated through direct contact with ligands released in their immediate vicinity through pH-dependent conformational changes [15]. OBPs from *Anopheles gambiae*, *Aedes aegypti and Culex quinquefasciatus* (AgamOBP1, AaegOBP1 and CquiOBP1 have been crystalised in dimeric form [15] [16] [17]. The crystal structures reveal a number of common structural features. The dimers are traversed by a single continuous hydrophobic channel, each end of which is flanked by an L-shaped side pocket open to bulk solvent. The dimeric interface is lined by residues belonging to helices 4 and 5 as well as a conserved Trp residue belonging to helix α6 of each subunit. A third opening to solvent is formed by residues of helices 4 and 5 at the dimer interface. In the X-ray structures of the AgamOBP1-DEET and AgamOBP1-6MH complexes, the ligands bind at exactly the same site in the periphery of the central binding cavity of each monomeric subunit close to the border of the dimer interface (see Fig 1) [18] [19]. DEET interacts through bridged hydrogen bonding with a conserved water molecule. No such interactions are observed for 6-MH despite the fact that the water molecule is also present at the same position in the crystallographic model. Similarly, in the highly homologous CquiOBP1 structure, two molecules of the mosquito oviposition pheromone (MOP) bind symmetrically in each subunit of the dimer with their respective lactone rings occupying part of binding site A of each subunit and their lipid tails extending into the hydrophobic tunnel of the dimer interface (see Fig 1) [16]. Another notable feature of the tertiary structure of these proteins is the position of the C-terminal loop of each subunit. This forms a “lid” over the binding pocket, which is held in place by a network of hydrogen bonds. It has been suggested that lowering the pH may disrupt the network of hydrogen bonds thus displacing the C-terminal loop from the binding pocket with a concomitant release of the bound ligands [15].

Whether these OBPs exist in monomeric or dimeric form under physiological conditions is a matter of debate. The exceptionally high concentration of OBPs (~10mM) in the sensillary lymph of insect antennae [20] [21] suggests that dimerisation may be physiologically relevant and thus conserved among mosquito OBPs [16] [17]. In gel filtration studies both monomeric and dimeric forms of CquiOBP1 have been isolated indicating the presence of a monomer-dimer equilibrium in solution [22].

In fluorescence-based competition binding assays of AgamOPBs 1 and 4, the synthetic repellent DEET and components of human sweat (indole, 6-MH) [23] have demonstrated that these compounds bind simultaneously with the fluorescent reporter 1-NPN [19]. In a recently solved X-ray structure, AgamOBP1 was shown to accommodate two molecules of the repellent Icaridin (*1-(1-methylpropoxycarbonyl)-2-(2-hydroxyethyl)piperidine*) [24]. Studies of other insect OBPs have also demonstrated that multiple ligand binding is possible [25] [26]. Interestingly, AgamOBPs 4 and 20 have been shown to undergo significant conformational transitions upon ligand binding [19] [27], which are deemed to be important in modulating the ability of these OBPs to form heterodimers. Heterodimerisation between different AgamOBPs that are co-expressed in the same antennal sensilla has been demonstrated in mosquitoes [28], as well as other insect species [29] [30]. The formation of heterodimers with binding properties that are different from those of the constituent monomers is thought to provide yet another mechanism of ligand discrimination [9] [31].

In this paper, we present molecular dynamics (MD) simulations of AgamOBP1 in complex with 6-MH and DEET to determine (a) whether simultaneous binding of two ligands brings about conformational transitions; (b) the effect, if any, of ligand binding on AgamOBP1 dimerisation, and (c) possible AgamOBP1 binding sub-sites that could be explored in the design of better analogues for ligand binding.

## Materials and methods

Crystallographic structures from the Protein Data Bank (PDB), PDB ID: 3N7H (AgamOBP1-DEET complex) and PDB ID: 4FQT (AgamOBP1-6MH complex) were used for docking and MD simulations.

### Docking simulations

Re-docking and cross-docking simulations were conducted using the Lamarckian genetic search algorithm (LGA) and the semi-empirical force field of the AutoDock (v.4.2) program [32]. The AutoDock Tools [32] GUI was used to remove crystallographic water molecules, with the exception of those buried in the binding pocket, and to add polar hydrogen atoms to proteins and ligands. The MMFF94 force field [33] was used for energy minimisation of the ligands. Ligand preparation involved the release of all torsions except those around conjugated double and triple bonds and Gasteiger partial charges were added while non-polar hydrogen atoms were merged. Protein preparation involved the addition of essential hydrogen atoms, Kollman united atom charges and solvation parameters. Proteins were covered, in their entirety, by affinity (grid) maps with spacing of 0.037 nm. The default parameters of AutoDock were used for distance-dependent dielectric functions, and van der Waals and electrostatic terms. The starting positions and orientation of the ligands were set randomly. Each ligand was subjected to 100–200 LGA runs of 5x10^6^ evaluations. The RMSD tolerance of the resulting docked structures was ≤ 0.2 nm. Docking results were sorted into bins of similar conformations. Cluster analysis or ‘structure binning” was performed based on all-atom root mean square deviation (RMSD). The resulting families of docked conformations were ranked in order of increasing energy (rank 1 was taken to be the lowest energy cluster).

### Molecular dynamics (MD) simulations

The AMBER12 and AMBERTools15 [34] programs were used for MD simulations and the analysis of the MD simulation data, respectively. Simulations were carried out in the isothermal isobaric thermodynamic ensemble at 300K using the ff99SB [35] and gaff [36] force-field parameters for the proteins and ligands respectively. Repeat simulations were carried out with AMBER 16 (ff14SB force-field parameters). The protein parameter and coordinate files were prepared using the LEAP module of AMBERTools15, whereas the corresponding ligand files were prepared using the Antechamber suite of AMBERTools. Acidic amino acids were treated in the fully protonated state and histidines were assigned protonation state +1. All complexes were charge neutralised with the addition of the requisite number of Na^+^ counter-ions using the LEAP module. Each system was immersed into a truncated octahedron periodic box containing water molecules. The TIP3P water model was used [37]. Periodic box boundaries were set at a distance of 0.9 nm from any solute atom.

The systems were first minimised using 500 steps of steepest descent minimisation followed by 500 steps of conjugate gradient minimisation using the Particle Mesh Ewald (PME) potential function and keeping the solute fixed. The harmonic restraint on the solute atoms was 840 kJ mol^-1^ nm^-2^. Restraints on solutes were removed and a second round of 1000 steps of steepest descent minimisation followed by 1500 steps of conjugate gradient minimisation was performed. The systems were heated from 0 K to 300 K by carrying out a 50 ps canonical ensemble (NVT)-MD during which harmonic restraints were applied to all solute atoms with force constants of 42 kJ mol^-1^ nm^-2^. Subsequently, the systems were subjected to 50 ps of isothermal isobaric (NPT)-MD at 300 K with coupling to Langevin thermostat. Constant pressure periodic boundaries conditions were applied with (a) isotropic scaling and 2.0 ps relaxation time to maintain the pressure at an average of 1 atm, and (b) weak restraints on the solute atoms (42 kJ mol^-1^ nm^-2^). Finally, the systems were equilibrated for an additional 2ns NVT-MD simulation at 300 K with a time constant of 2.0 ps for Langevin bath coupling and removal of the constraints on the solute. Equilibration of the system was followed by production time MD for each of the complexes. All MD simulations were carried out with constraints on hydrogen atoms using the SHAKE algorithm [38], 0.8 pm electrostatic interactions cut off using the PME method and a time step of 2 fs.

AMBERTools17 [34] and Chimera [39] were used for trajectory analysis and molecular visualisation. Principal components analysis (PCA) of distributions of protein conformations as a function of time was conducted using the Bio3D tool [40].

### Constant pH MD simulations

The Amber 16 package was used for the simulations. Topologies were prepared using leaprc.constph, which loads the ff14SB force field together with special carboxylate residue libraries that define a hydrogen atom at each protonable location and sets GB solvation radii. The water model used was TIP3P and starting structures of each simulation were solvated in truncated octahedron box with 0.9 nm solute-wall distance. Energy minimisations with restraints on the protein backbone and constant pH specified were followed by heating the structure from 0 to 300 K over 400 ps at constant volume. Prior to production MD, the systems were stabilised by running the NPT ensembles for 4ns.

### MM-GBSA

Molecular Mechanics Generalised Born Surface Area (MM-GBSA) calculations were performed to elucidate the thermodynamic properties of the systems and, in particular, to predict relative protein-protein and protein-ligand binding affinities. The calculations were performed on the last 40 ns of the MD simulation time using the MMPBSA.py module [41] of AmberTools15 using the GB model developed by Onufriev et al. [42]. Exterior dielectric constant of 80 and solute dielectric constant of 1 were used. This single trajectory method was used [43]. The method is deemed to be accurate enough to compare relative free energies of binding of different ligands to the same receptor [44] [45].

## Results and discussion

To ascertain whether the presence of DEET or 6-MH has an effect on the formation of the AgamOBP1 homodimer, we conducted MD simulations of the dimeric form of the protein in the presence and absence of the ligands, measuring the relative binding energies by means of MMGBSA.

### MD simulations of dimeric complexes: time-dependent properties

**Fig 2:**
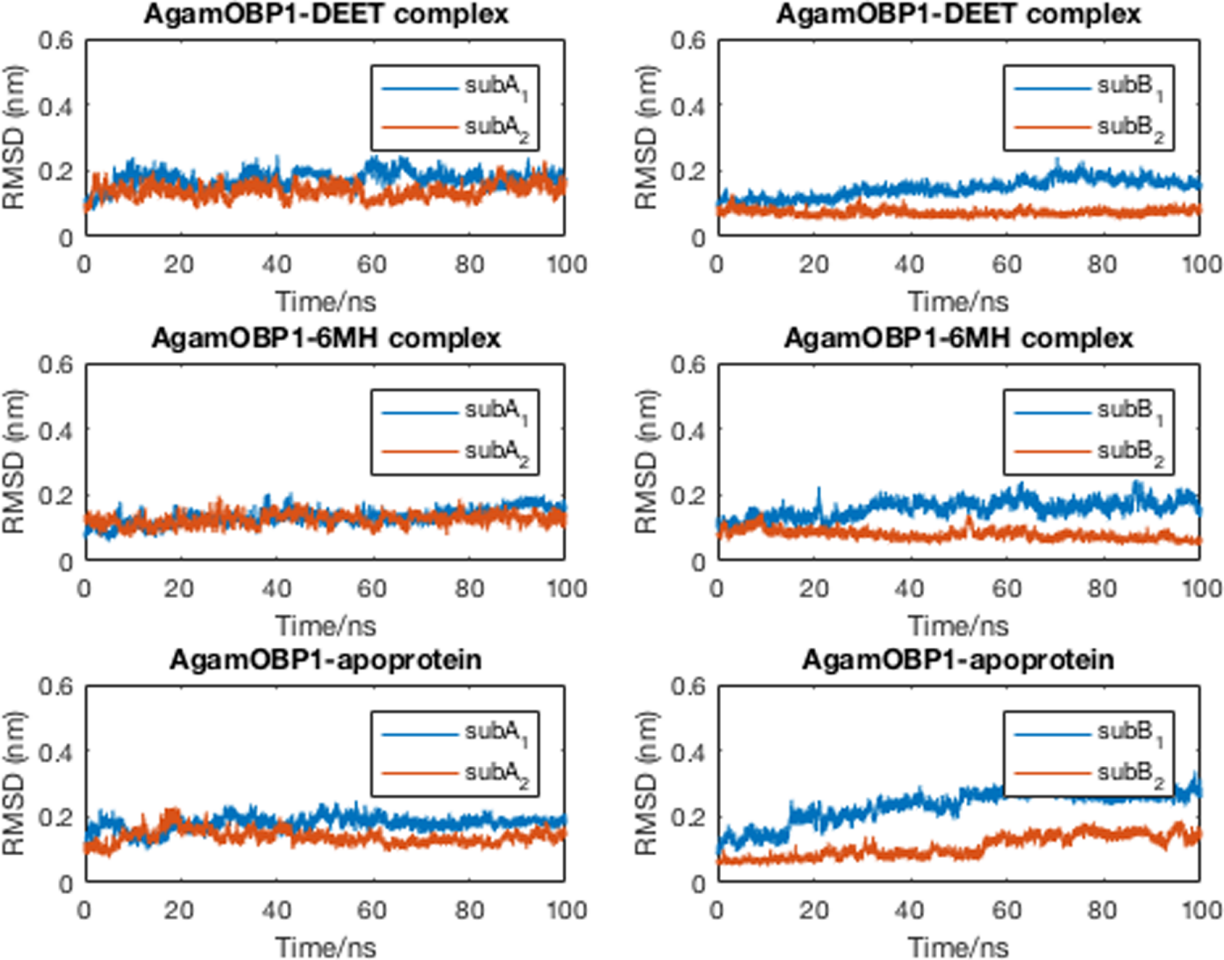
Time-series of RMSD of backbone atoms of AgamOBP1 from the starting structures over 100 ns of MD simulations. (a) AgamOBP1 chain A in complex with DEET and 6-MH, and in the absence of ligand; (b) similarly for chain B. Subscripts 1 and 2 indicate different sets of MD simulations.

The starting coordinates for the MD simulations were taken from the X-ray structures of AgamOBP1-DEET (PDB ID: 3N7H) and AgamOBP1-6MH (PDB ID: 4FQT) and unliganded AgamOBP1 (PDB ID: 3N7H). Two sets of simulations were carried out with the PME method in explicit water for 100ns. For the liganded complexes, the crystallographically conserved water molecule was included as part of the solute. The RMSD values of the protein backbone atoms remained below 0.25 nm for the course of the simulations, with the exception of chain B of the unliganded protein (S1 Table). The stability of the protein during the simulations is illustrated in Fig 2 as the time series of the RMSD of backbone atoms from the starting structure.

**Fig 3:**
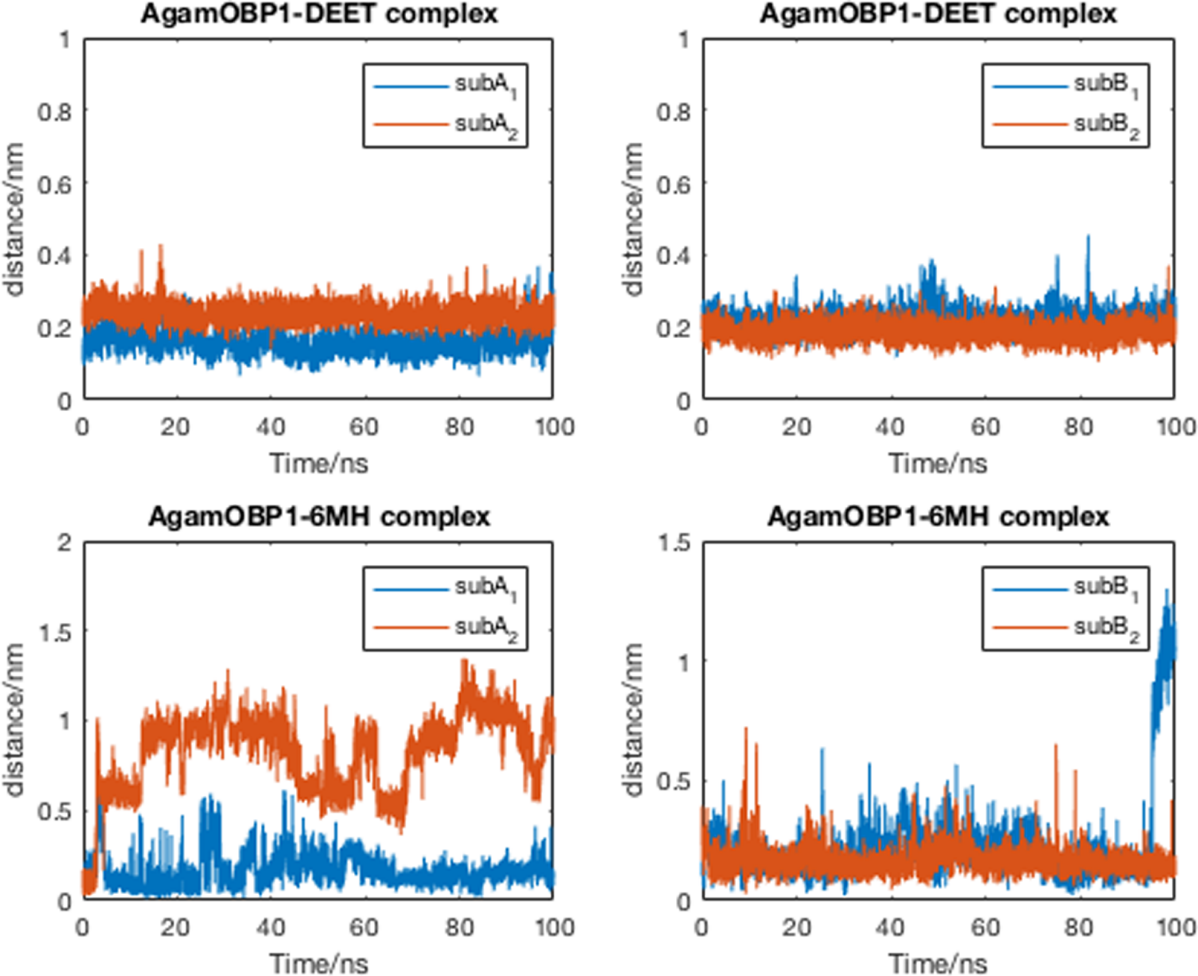
Distance between the centre of mass of the ligand and that of residues lying within 0.3 nm at the start of the simulation. Subscripts 1 and 2 indicate different sets of MD simulations.

In the course of the simulations, DEET formed bridged hydrogen bonds involving the crystallographically conserved water molecule and residues Cys95 and Trp114 (occupancy of ~93% for chains A and B) remaining anchored in the vicinity of these residues. In the case of 6-MH, in both simulations, the ligand formed transient hydrogen bonds (< 5% occupancy) and moved away from its original binding site. Fig 3 illustrates the relative mobility of the ligands within the binding pocket of the protein.

**Fig 4:**
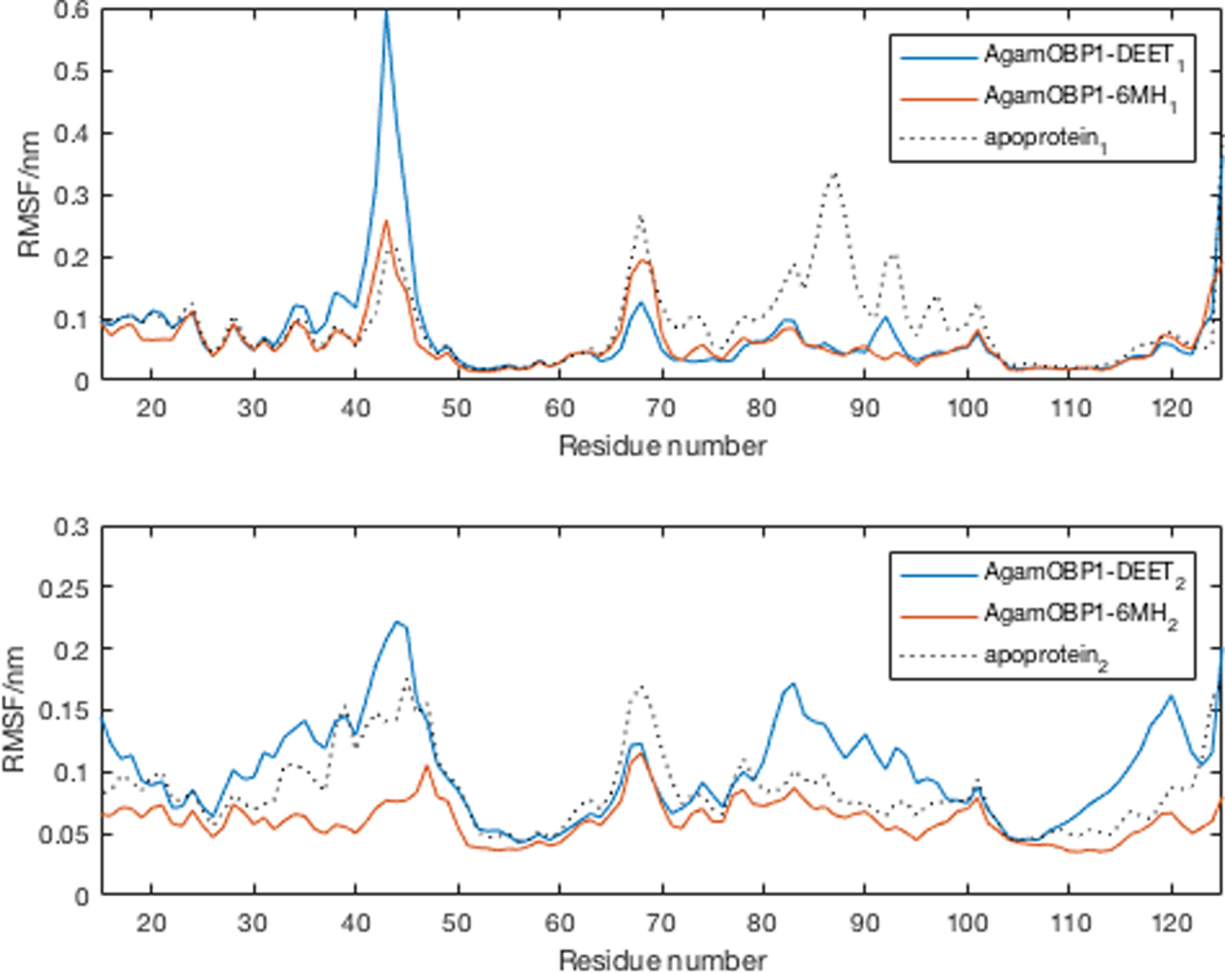
RMSF of backbone atoms of subunit A of the AgamOBP1 apoprotein as well as in complex with DEET and 6-MH. Subscripts 1 and 2 indicate different sets of MD simulations. Subscipts 1 and 2 are first and second sets of simulations.

Conformational variance was measured by root mean square fluctuations (RMSF) of the protein α-carbon atoms (C_α_) of the complexes [46]. The parts of structure that fluctuate the most from their mean structure are shown in Fig 4. In both complexes the largest displacements are observed between residues 35–45 (helix 2-bend), 66–71 (anti-turn-bend-anti) and 85–102 (helix5-turn-bend). A similar overall pattern of RMSF fluctuations was observed in the case of the AgamOBP1 apoprotein.

Principal component analysis (PCA) [47] shows that the first four principal components capture ~50% of the sampled large-scale protein motions during the trajectories (S1–S3 Figs) with most of the structural variation occurring in the same regions as those depicted in Fig 4. Secondary structure content analysis of the complexes as a function of time using the DSSP method of Kabsch and Sander [48] revealed no significant changes in secondary structure of AgamOBP1 upon complexation with DEET and 6-MH (S4–S6 Figs).

### Time-independent properties: binding energy calculations

**Fig 5:**
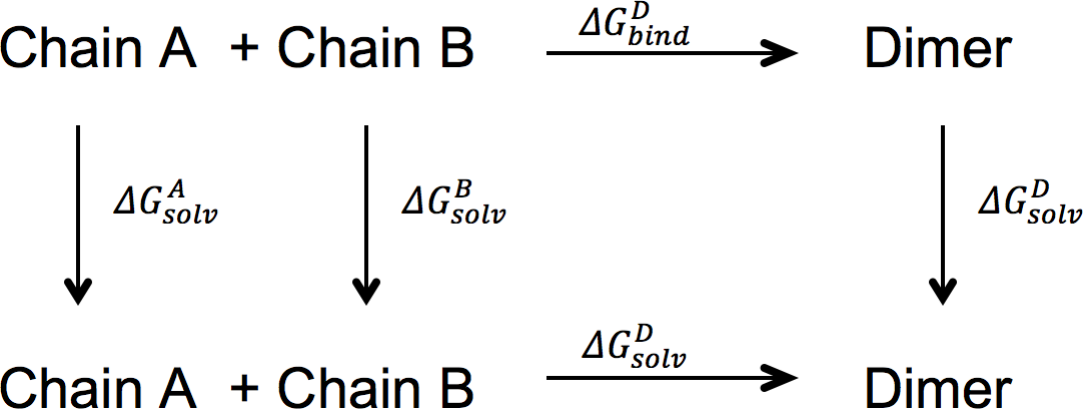
Thermodynamic cycle for the calculation of the binding free energies between chains A and B of AgamOBP1 in the gas phase, $\Delta G_{bind}^{o}$, and in solution $\Delta G_{bind}$. The solvation free energies of chain A, chain B, and of the dimer are $\Delta G_{solv}^{A}$, $\Delta G_{solv}^{B}$, and $\Delta G_{solv}^{D}$, respectively. For the DEET and 6-MH complexes, chain A = chain A + ligand and chain B = chain B + ligand.

Assuming the presence of a monomer-dimer equilibrium in solution [22], the effect of ligand binding on shifting the equilibrium in either direction can be studied by estimating the “effective energies” of binding of the two putative subunits of AgamOBP1 in the presence and absence of ligands. “Effective energies” of binding (ΔG_gas+solv_) of the two subunits of the AgamOBP1 dimer were calculated, in the presence and absence of ligands using the method described in Miller *et al*. [41]. The thermodynamic cycle used to calculate the binding energies between chain A and chain B of the protein is shown in Fig 5.

Although “effective energies” do not include the entropic component of absolute free energies, they are a reliable comparator of binding affinities [49]. Solvation free energies were calculated at an ionic strength of 10 mM. We report the effective energies of binding of the two subunits of AgamOBP1 in the presence and absence of DEET and 6-MH (Table 1). We extended our studies to the AgamOBP1-Icaridin complex to ascertain any similarities and or difference in the binding with regard to DEET and 6-MH.

**Table 1:** “Effective energies” of binding of the two subunits of the AgamOBP1 dimer apoprotein and in complex with DEET, 6MH and Icaridin obtained from two independent sets of 100 ns MD simulations each (AMBER12 first row, AMBER16 second row).

Receptor	AgamOBP1_[A:B]_		AgamOBP1_[A:B]_		AgamOBP1_[A:B]_		AgamOBP1_[A:B]_	
Ligand	DEET		Icaridin		6-MH		none	
	Δ value^a^	σ^b^	Δ value^a^	σ^b^	Δ value^a^	σ^b^	Δ value^a^	σ^b^
***ΔG***_***gas+solv***_	-204.4	34.7	NA	NA	-157.2	30.1	-116.1	24.4
***ΔG***_***gas+solv***_	-238.2	31.3	-239.4	23.0	-204.8	20.3	-181.1	28.2

^**a**^Average difference (Complex-Receptor-Ligand)

^**b**^Standard deviation. Energy values in kJ mol^-1^

The results summarized in Table 1 indicate that complexation with DEET, Icaridin, and to a lesser extent 6-MH, favours the interaction of the monomeric subunits of the protein. The relative contributions of the enthalpic and solvation components to *ΔG*_*gas+solv*_ are given in S2 and S3 Tables, where it is shown that the enhanced free energy of interaction of the two subunits in the presence of ligands is mainly due to the enthalpic (ΔH_gas_) and non-polar solvation (ΔG_np_) components of ΔG_gas+solv_ as compared to the corresponding terms of the unliganded dimer. Per-residue decomposition of the free energies of binding [50] shows that Icaridin, DEET and 6-MH contribute -34.0, -30.4 and -17.4 kJ/mol to the two subunits of AgamOBP1 (S4 Table). Pairwise per-residue decomposition of free energies of binding shows that the two DEET and Icaridin molecules bound to each subunit interact positively with each other, as well as with Met89, Lys93, Arg94 and Leu96 of the opposite subunit. No such interactions were observed in the case of 6-MH (S5 Table).

Analysis of the “effective” free energies of binding of the ligands to AgamOBP1 shows that Icaridin and DEET have considerably higher affinity to the protein than 6-MH due in part to their higher molecular mass, as well as to the specificity of their interactions with AgamOBP1 (Table 2). The relative contributions of the enthalpic and solvation components to *ΔG*_*gas+solv*_ are given in S6 and S7 Tables.

**Table 2:** “Effective” energies of binding of DEET, Icaridin and 6-MH to AgamOBP1 (subunits A, B).

		AgamOBP1-DEET		AgamOBP1-Icaridin		AgamOBP1-6-MH	
	No. MD	ΔG_gas+sol_	σ	ΔG_gas+sol_	σ	ΔG_gas+sol_	σ
**Subunit A**	1	-134.4	8.9	NA	NA	-93.2	7.5
	2	-128.4	8.6	-170.5	9.4	-71.2	9.3
**Subunit B**	1	-136.0	8.3	NA	NA	-85.0	10.8
	2	-134.9	8.5	-154.9	11.5	-95.6	7.6

σ = Standard deviation. Energy values in kJ mol^-1^. **No. MD** is number of independent simulation.

### Multiligand complexes

**Fig 6:**
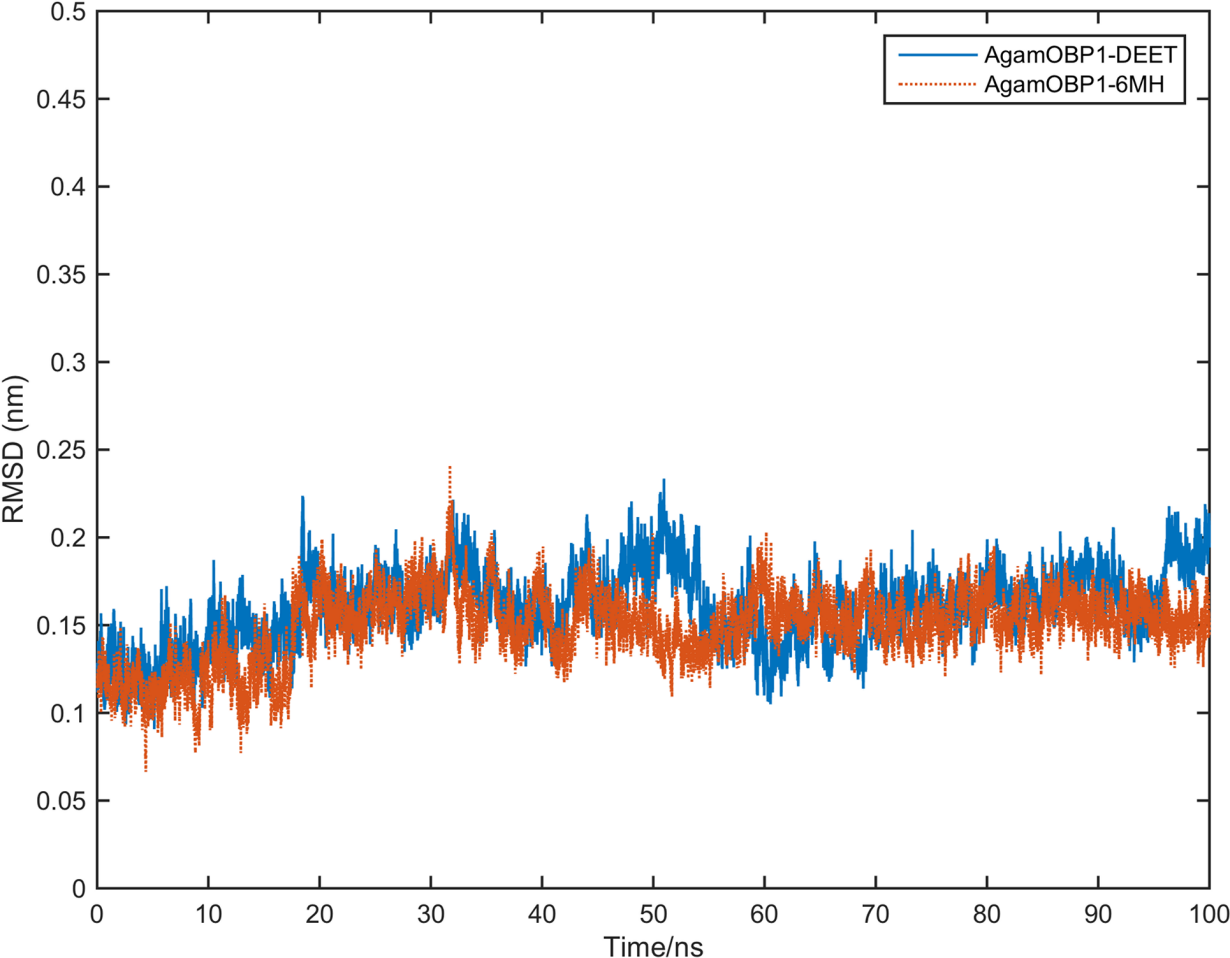
Protein backbone RMSD time series of AgamOBP1 multiligand complexes.

To study the presence or absence of allosteric effects upon simultaneous binding of two ligands we used the crystallographic structures of chain A of AgamOBP1 (PDB ID: 4FQT) in complex with DEET and 6-MH. We refer to these as AgamOBP1-DEET_[X-ray]_ and AgamOBP1-6MH_[X-ray]_, respectively. These structures were used for the docking of a second ligand molecule, DEET or 6-MH as appropriate. All water molecules but the one that is conserved in both crystallographic models were stripped, together with all ions and serendipitous ligands. In both cases (DEET and 6-MH), the lowest energy clusters of ligands were docked in the central cavity of AgamOBP1. Representative structures from the lowest energy clusters were used in MD simulations. We call these complexes AgamOBP1-DEET_[X-ray]_-DEET_[docked]_ and AgamOBP1-6MH_[X-ray]_-6MH_[docked]_, respectively. Subsequently, the complexes were subjected to minimisation, equilibration and 100ns production MD simulations using the same parameters as those described for the dimers. The range of RMSD of the protein Cα atoms in the respective X-ray structures is given in S8 Table. RMSD fluctuations of the protein Cα atoms with respect to the initial structures over the simulation period are shown in Fig 6.

**Fig 7:**
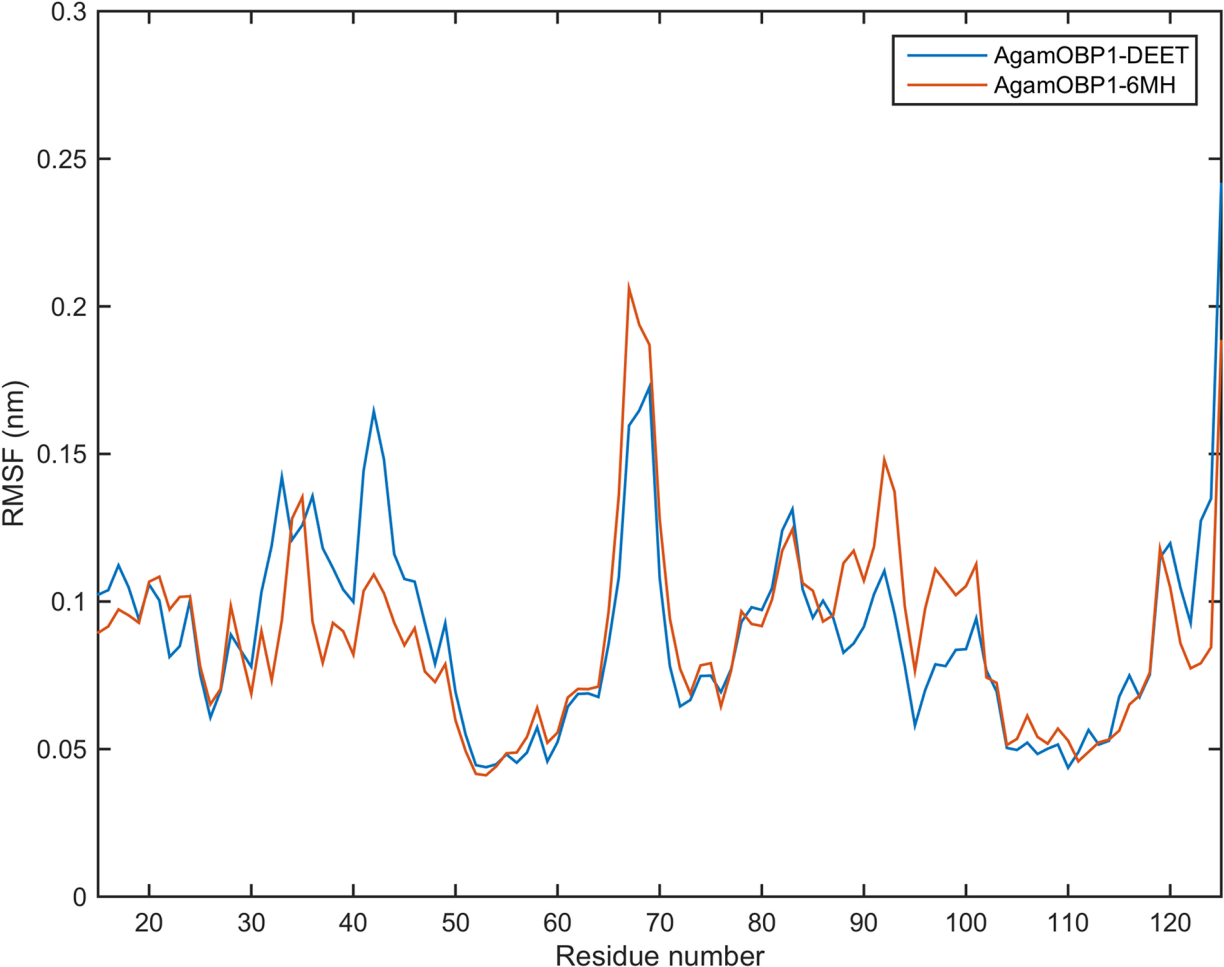
RMSF of backbone atoms of AgamOBP1-DEET and AgamOBP1-6MH multiligand complexes.

The average structures obtained from the trajectories were almost identical (backbone RMSD = 0.05 nm). As shown in Fig 7, the RMSF profiles of the two structures were very similar to those described for the dimers (cf. Fig 5).

Similarly, secondary structure analysis (S7 and S8 Figs) and principal components analysis (S9–S11 Figs) showed that most structural variations were in the same regions as those obtained from the MD trajectories of the dimers (cf. S1, S2 and S3 Figs). The results obtained suggest that the presence of the second ligand does not induce any major conformational changes in the 3D structure of AgamOBP1. DEET_[X-ray]_ behaved similarly to the dimeric complex and remained bound at the periphery of the central cavity of AgamOBP1. It was within van der Waals distance of residues of the 4th and 5th helices and formed bridged hydrogen bonds with the solvent and residues Trp114 and Cys95 for almost 90% of the simulation time. DEET_[docked]_ remained bound in the central cavity of the protein making contact with C-terminal residues (His121, Y122, Phe123) as well as forming bridged hydrogen bonds with water, Met55 and His111 for a significant part of the simulation time (Table 3).

**Table 3:** Distance between ligand and receptor residue atoms closer than 0.36 nm.

Ligand	Contact residue (TotalFrac, Contacts)	H-bonds
DEET_[X-ray]_	L73 (2.02, 4), L76 (2.43, 5), H77 (3.60, 13), L80 (0.05, 1), A88 (3.57, 8), M89 (0.15, 2), M91 (0.14, 1), G92 (1.55, 5), L96 (0.10, 3), W114 (3.26, 7), DEET_[docked]_ (0.07, 3)	DEET@O1—HOH bridged with Trp114, CYS95 (Frac. ~86%)
6-MH_[X-ray]_	L76 (0.20, 2), H77 (0.06, 3), L80 (0.12, 1), A88 (1.13, 5), M91 (0.06, 1), G92 (0.02, 2), W114 (0.04, 1)	6-MH@O—HOH (Frac. ~1.4%); 6-MH@O—Phe123@N (Frac. < 1%)
DEET_[docked]_	Y10 (0.10, 1), L15 (0.04, 1), L80 (0.04, 2), M84 (0.15, 4), A88 (0.00, 2), M89 (0.04, 5), M91 (0.04, 5), H111 (0.04, 3), W114 (0.06, 2), H121 (0.018, 3), Y122 (0.02, 7), F123 (0.54, 16), L124 (0.00, 2), DEET_[X-ray_ (0.07, 3)	DEET@O1—HOH bridged with His111, Met55 (Frac. ~16%)
6-MH_[docked]_	H111 (0.11, 2), W114 (0.00, 1), Y122 (0.00, 1), F123 (0.50, 17), L124 (0.01, 6)	6-MH@O—His111 (Frac. ~1.5%); 6-MH—Phe123@N (Frac. < 1%); 6-MH—Ser79@O (Frac. < 1%)

**Contacts** is the total number of contacts per ligand residue pair: **TotalFrac** is the sum of each contact involving that pair divided by the total number of frames. Total number of frames is 4,000 (last 40 ns of the simulation time)

**Fig 8:**
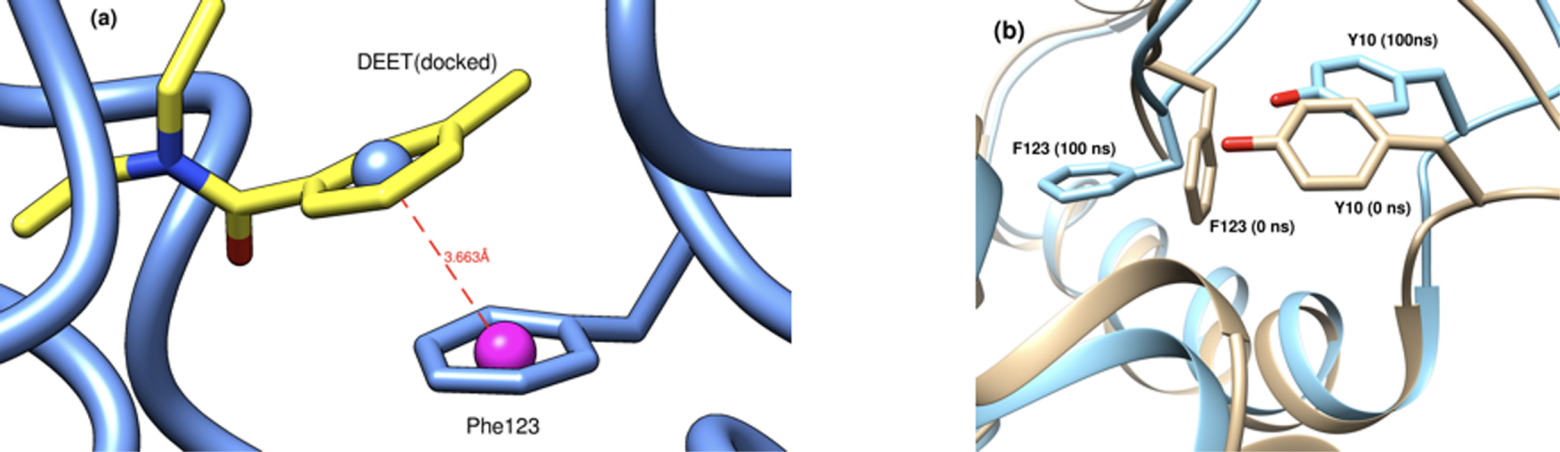
(a) π-π interactions between the aromatic rings of DEET and Phe123. (b) relative positions of Tyr10 and Phe123 at the start and end of the MD simulation (the angle between the ring planes of DEET and Phe123 is 19.2º). In the crystallographic model the phenyl ring of Phe123 lies perpendicular to the plane of the aromatic ring of Tyr10 (91.3° for the A and 89.3° for the B chains, respectively). It rotates by ~ 70° to form stacking interactions with the aromatic moiety of DEET.

Interestingly, DEET_[docked]_ is stabilised in the binding site by face-to-face π-π stacking interactions with Phe123 [51]. The side chain of Phe123 rotates by approximately 90° with respect to its crystallographic conformation to form two-ring stacking interactions with the aromatic moiety of DEET (Fig 8).

Additionally, the backbone NH or carbonyl of Phe123 may also be involved in direct H-bonding (see Table 3 above). The same finding has been reported in studies involving the highly homologous *Aedes aegypti* AaegOBP1 and a number of small ligands [52] and is also confirmed by independent docking and MD simulations involving AgamOBP1 and 6-methyl-2-(4-methylcyclohex-3-en-1-yl)hept-5-en-2-ol (bisabolol) [26] or (2E)-3,7-dimethylocta-2,6-dien-1-ol (geraniol) (paper in preparation).

**Fig 9:**
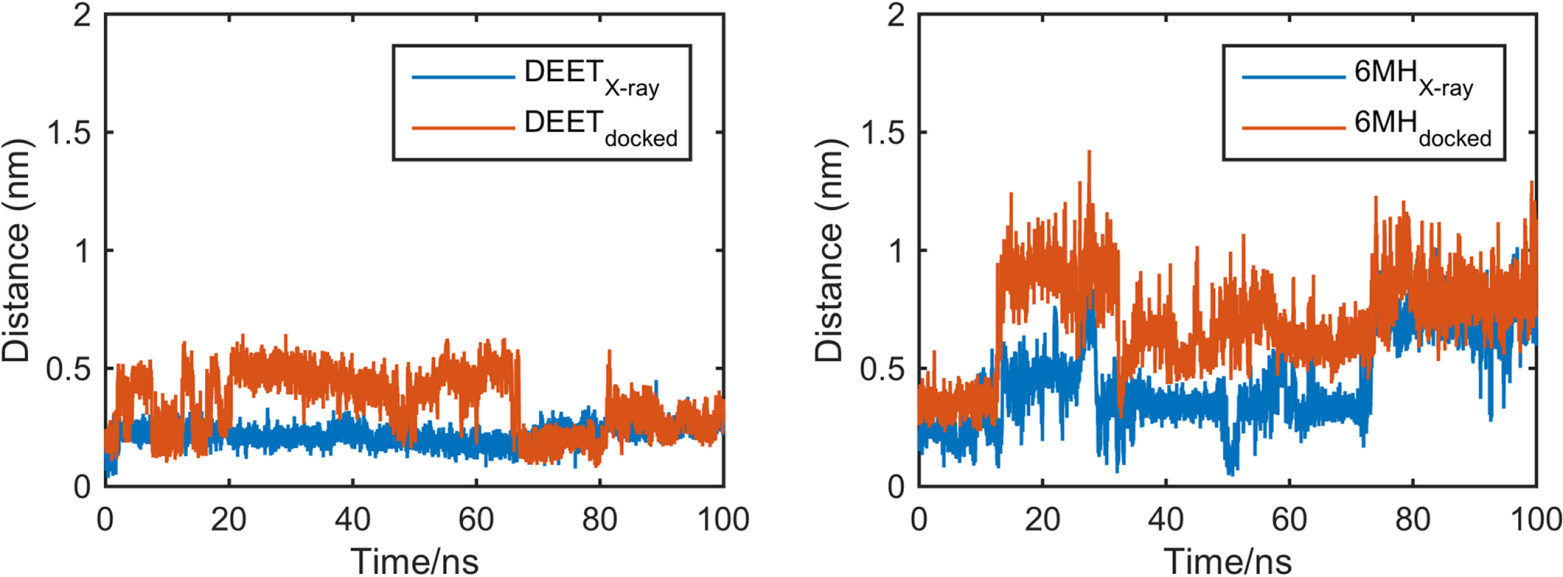
AgamOBP1-multiligand complexes. Distance between the centre of mass of the ligand and that of residues lying within 0.30 nm at the start of the simulation.

**Fig 10:**
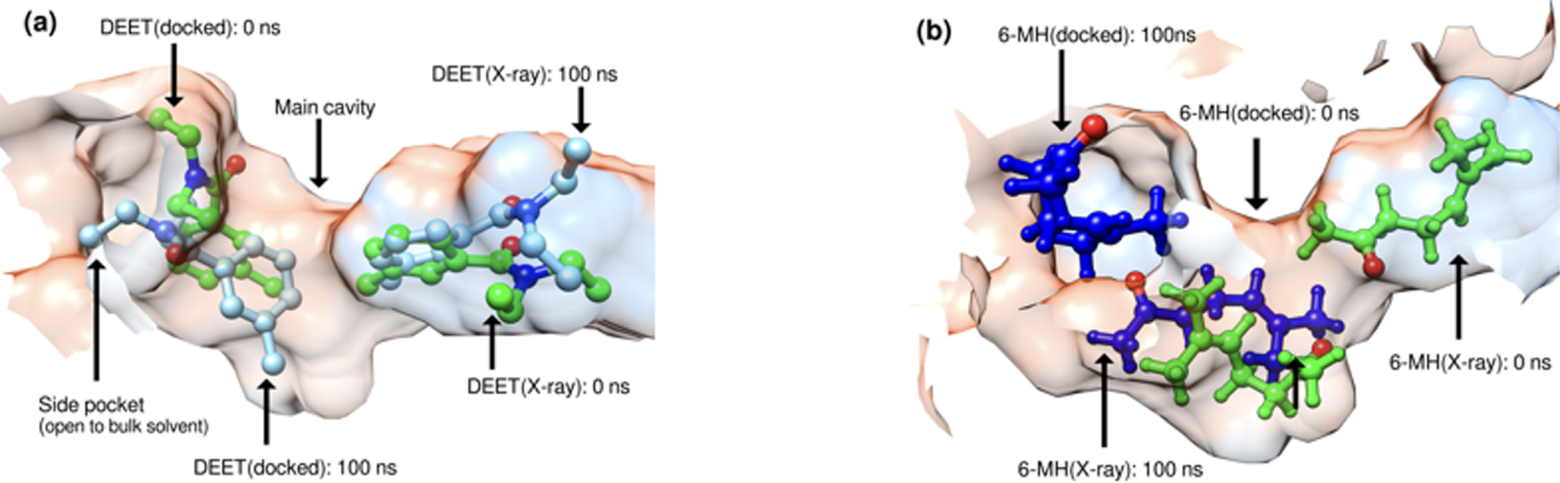
AgamOBP1-multiligand complexes. Relative positions of ligands at the start and end of the MD simulations (100 ns). (a) AgamOBP1-DEET; (b) AgamOBP1-6MH. DEET_[X-ray]_ and 6-MH_[X-ray]_ are the evolution of ligand X-ray structures. Correspondingly, DEET_[docked]_ and 6-MH_[docked]_ are the evolution of ligand docked structures.

In contrast to DEET, the MD simulations did not reveal any specific interactions between 6-MH and the receptor. In the course of the simulation, 6-MH_[X-ray]_ was shown to move away from its initial position towards the central cavity of the receptor displacing 6-MH_[docked]_. The latter moved to the adjacent side pocket (see Fig 1) and out of it. Figs 9 and 10 depict the movement of the ligands during the trajectories.

### Time independent properties

The calculated free energies of binding of ligands are shown in Table 4.

**Table 4:** “Effective energies” of binding of AgamOBP1 DEET and 6MH multiligand complexes.

Ligand	DEET_X-ray_		DEET_docked_		6-MH_X-ray_		6-MH_docked_	
	ΔG_gas+solv_	σ	ΔG_gas+solv_	σ	ΔG_gas+solv_	σ	ΔG_gas+solv_	σ
	-112.1	11.0	-90.5	9.1	-84.5	11.2	-72.3	8.2

σ = Standard deviation; Energy values in kJ mol^-1^; DEET_X-ray_ / 6-MH_X-ray_ are ligands in the conformation of the X-ray models at the start of the simulations; DEET_docked_ / 6-MH_docked_ are ligands in the conformation of the lowest emerged docked models at the start of the simulations. See also S9 Table.

The binding affinity of DEET_X-ray_, as compared to DEET_docked_, is due mainly to the bridged hydrogen bonds involving the ligand that result in considerably more favourable ΔH_elec_ than that of DEET_docked_. The gain in ΔH_elec_ more than compensates the higher penalty in electrostatic desolvation (ΔG_GB_) upon complex formation. It is also noted that solvent accessible surface area (SASA) of DEET_X-ray_ is considerably larger than that of DEET_docked_ (SASAs for the starting structures of the MD simulations: 0.78 nm^2^ and 0.24 nm^2^, respectively). Correspondingly, a comparison of the binding affinities between DEET_X-ray_ in the multiligand complex and DEET in the dimeric complex (cf. Table 2) shows that the higher binding affinity of the latter is almost exclusively due to its more favourable ΔG_GB_ (cf. SASA of DEET in the dimer of 0.10 nm^2^). By analogy, in the case of 6-MH the relatively higher binding affinity of 6-MH_X-ray_, as compared to that of 6-MH_docked_, is mainly due to the enthalpic component of ΔG_gas+solv_ that compensates the penalty in ΔG_GB_. As in the case of DEET, SASA of 6-MH_X-ray_ is considerably larger than that of 6-MH_docked_ (0.42 nm^2^ and 0.15 nm^2^, respectively).

Pairwise per-residue free energy decomposition showed that the two ligand molecules in each complex do not impose steric constraints on each other (pairwise ligand-ligand energy decomposition = ~ -4.2 kJmol^-1^). Other interactions between the ligands and protein residues are shown in S10 Table.

### Constant pH MD (CpHMD) simulations at acidic pH

In order to test whether lowering the pH to 5 has an effect on the AgamOBP1 protein structure, we conducted CpHMD simulations. It has been proposed that the low pH at the vicinity of the dendritic membrane disrupts the network of hydrogen bonds holding the C-terminal loop from the binding pocket resulting in the release of the bound ligands [15]. The advantage of CpHMD is that it leverages the ability of conventional MD by sampling at the same time both the conformational space and the available protonation state distributions [41].

Asp, Glu and His were titrated, resulting in 34 titrable residues the protonation states of which are given in S11 Table. We conducted three separate 20 ns simulations each in the presence of Icaridin at pH5. A further 20 ns simulation was performed for the unliganded AgamOBP1 monomer (PDB ID: 5el2, chain A). RMSD fluctuations of the protein Cα atoms with respect to the crystal structure over the simulation periods are given in S12 Fig together with the RMSF profiles of the structures at pH7 and pH5, S13 Fig.

The titrable residues involved in hydrogen bonds holding the “lid” in place as well as their protonation states at pH5 and 7 are shown it Table 5. Although, the changes in protonation state that occur at pH5 result in lower hydrogen bond occupancies, they do not suffice to disrupt sufficiently the network of hydrogen bonds. As a result, the C-terminal “lid” was held in place during the simulations.

**Table 5:** Protonation states and hydrogen bonds of residues maintaining the integrity of the C-terminal loop.

Residue	pH	Offset	Pred	FracProt	H-bonds	%	H-bonds	%
Asp7	7	-inf	-inf	0	Asp7-Arg5	96.2	Asp7-Tyr10	8.5
	5	-1.041	3.959	0.083		100		58.6
Asp42	7	-2.25	4.75	0.006	Asp42-Arg6	8.2		
	5	-1.334	3.666	0.044		1.8		
Asp118	7	-2.219	4.781	0.006	Asp118-Lys20	0.0		
	5	-2.321	2.679	0.005		0.0		
His121	7	-0.474	6.526	0.251	His121-Asp118	43.0		
	5	1.248	6.248	0.946		100.0		
His23	7	1.583	8.583	0.975	His23-Val125	96.9	His23-Tyr54	56.0
	5	1.944	6.944	0.989		67.8		50.5
Val125	7				Val125-Tyr54	11.0		
	5					4.7		

**Offset:** is is the difference between the predicted pK_a_ and the system pH; **Pred:** is is the predicted pK_a_; **FracProt:** is the fraction of time the residue spends protonated; **%:** is the occupancy of hydrogen bonds

**Fig 11:**
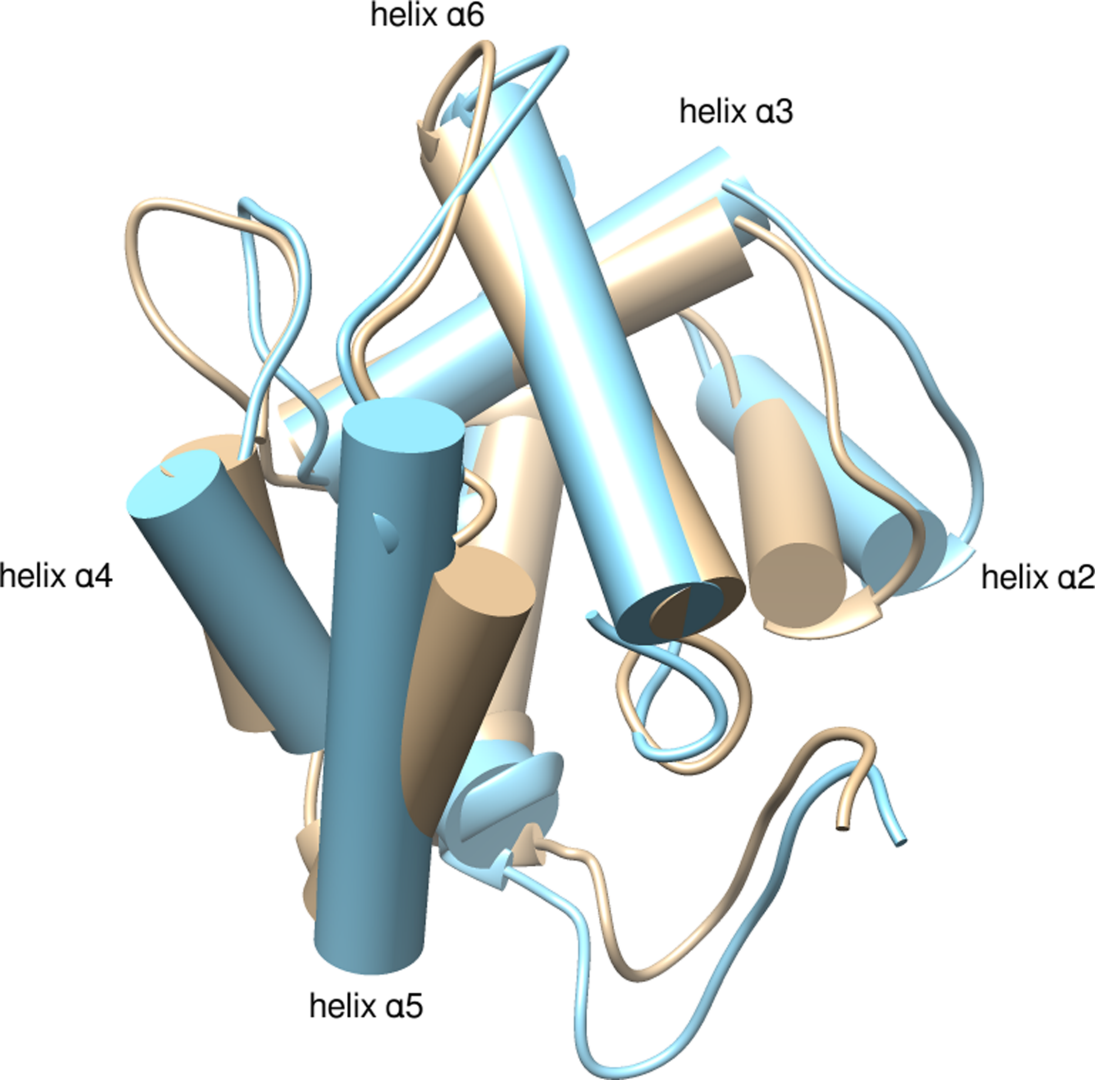
pH-induced conformational changes of AgamOBP1 (cyan depicts the conformation at pH5).

The secondary structure of the protein remained mostly intact at pH5 with the exception of the first 5 residues of helix α1, which underwent a conformational change to β-turn for part of simulations (S14 Fig). At pH5, we did, however, observe notable changes in the conformation of the loop between helices α3 and α4 (residues 63–72) and in the angles between helices α2- α3 and α4- α5. The average tilt of these angles with respect to the corresponding ones of the crystal structure was in the range of 12° to 15° (Fig 11). We note, however, that the angle fluctuations were large indicating that the systems had not converged to stable values. In all simulations of the AgamOBP1-Icaridin complex, the ligand was not released from the binding site in the course of the simulations.

## Discussion

### Simultaneous binding of ligands and effects on allostery

**Fig 12:**
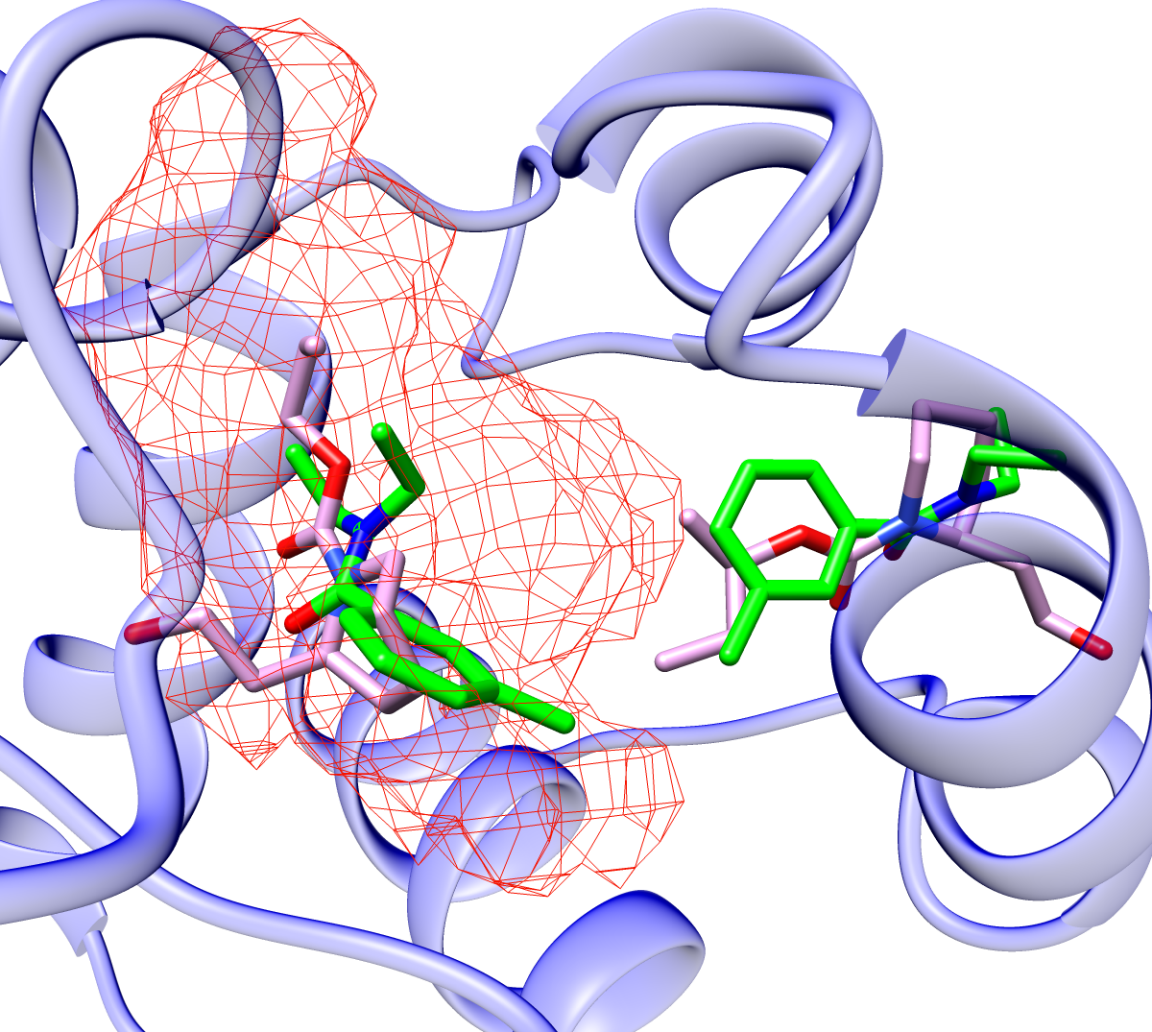
AgamOBP1 binding sites for the two molecules of Icaridin (PDB ID: 5EL2) (magenta). DEET_[X-ray]_ and DEET_[docked]_ in green. The grid circumscribes all conformations adopted by DEET[docked] during the trajectory.

Our computational simulations show that ligand binding can occur in the central cavity of the monomeric subunit of AgamOBP1 as well as proximally to the dimeric interface of the putative AgamOBP1 dimer. This is consistent with very recent structural studies [24] that provide evidence of binding of the repellent Icaridin to AgamOBP1 dimer at the same sites that were predicted by the MD simulations for AgamOBP1-DEET multiligand complex reported in this paper (Fig 12). Similarly, to DEET, one molecule of Icaridin was bound at the binding site formed by helices 4, 5 and Trp114 making the very same bridged hydrogen bonding interactions with Trp114 and Cys95. The second Icaridin molecule was shown to interact by means of bridged hydrogen bonding with Met55 and His111.

The results of our docking simulations directed at the hydrophobic channel formed between helices 4 and 5 are consistent with similar studies involving AgamOBP1 [53] as well as the highly homologous CquiOBP1 [54]. Ligand binding at the central cavity of AgamOBP1 is also possible as shown in simulations in which the docking grid was centred at it. Our MD simulations and MMGBSA calculations show that DEET is stabilised through polar interactions (bridged hydrogen bonding), as well as favourable van der Waals interactions of its diethyl moieties with residues of helices 4 and 5. Ligands, such as 6-MH, bound at the hydrophobic channel and lacking such stabilising interactions may be free to migrate towards the central cavity of AgamOBP1 unless the latter is occluded by another ligand of the same or higher binding affinity.

We did not observe any allosteric effects upon single or multiligand binding. However, in multiligand binding, steric crowding constrains one of the two ligands into the space proximal to the carboxy-terminus of the protein. This is reflected in Table 3, which shows that His111 and the residues of the carboxy-terminus (Tyr122, Phe123 and Leu124) are in contact with the ligands for a significant fraction of the MD simulation time. Our conclusion that these residues may play an important role in ligand binding and/or ligand specificity is in agreement with results obtained from site-directed mutagenesis and modelling studies [55]. Whether or not multiligand binding is required for biological activity remains a moot point. Furthermore, ligand binding at two distinct sub-sites of the binding pocket of the protein raises the issue whether ligand uptake takes place sequentially through a single gate or through different protein gates.

### Effect of ligand binding on the dimerisation of AgamOBP1

The biological relevance of the dimeric form of AgamOBP1 has been the matter of some debate. In the first reported X-ray structure of the AgamOBP1 dimer in complex with the serendipitous ligand polyethylene glycol (PEG), Wogulis et al. [17] suggest that due to the large percentage of polar contacts in the dimer interface, the protein is likely to be a monomer in the antennae; however, they also point out that the protein may exist as a dimer if its concentration in the sensillum lymph is sufficiently high. In a subsequent paper on the crystal structure of AgamOBP1 in complex with DEET, Tsitsanou et al. [18] suggest that since a monomer-dimer equilibrium has been shown to exist in solution [16] [22] and the concentration of AgamOBP1 in the sensillum lymph is likely to be exceptionally high by analogy to other OBPs [21], it is possible that the dimer is the molecular target of DEET under physiological conditions [18]. However, in NMR relaxation studies, it was shown that AgamOBP1 is dimeric in the absence of ligands [56] but monomeric in the presence of DEET and/or 6-MH and that these repellents bind at the same site close to the interface of the two putative subunits of the dimer [19]. Based on evidence that AgamOBP1-OBP4 heterodimers in complex with components of the human sweat such as indole and 3-methy indole are important for odour perception, the authors suggest that binding of repellents such as DEET and 6-MH may either compete and/or displace the normal odourant (e.g. indole) thereby disrupting the formation of the AgamOBP1-OBP4 heterodimers.

Our MD simulations of the monomeric species of AgamOBP1 establish that DEET and Icaridin form specific bridged H-bond interactons with the receptor and bind at the same location as that reported in the literature [18] [24], and as such may disrupt the formation of the AgamOBP1-OBP4 heterodimer as proposed by Murphy et al. [19]. In the case of 6-MH, interactions between the ligand and AgamOBP1 are non-specific and the ligand is shown to migrate towards the central binding pocket in the absence of a second ligand which could occlude it.

However, in the light of our MMGBSA calculations and assuming the presence of a dynamic equilibrium between the monomeric and dimeric states of AgamOBP1 (see Fig 5), we cannot exclude the possibility that initial binding of these repellents to the AgamOBP1 monomer may shift the equilibrium in favour of the dimeric state. In this case, homodimerisation could effectively deplete the number of AgamOBP1 monomers available to form AgamOBP1-OBP4 heterodimers and thus block or attenuate odorant response to components of human sweat. The differences in the conditions employed in the in-vitro experiments of Murphy et al. [19] and the in-silico simulations reported in this paper (e.g. time scales, buffer composition) could lead to a predominance of monomer in the in-vitro experiment due to the equilibrium position in low concentration solutions or a slow rate of achieving equilibrium. We propose that these two alternative mechanisms of repellent action may not be mutually exclusive and could, therefore, function in tandem.

### Effect of pH on conformational stability

Our results are in agreement with a previous CpHMD study involving the orthologous CuiOBP1-MOP complex [57], in which it was shown that the C-terminal “lid” remains attached to the main binding cavity of the protein at low pH 4.5. The same study also revealed significant conformational changes involving the loop between helices α3 and α4, as well as helices α4-α5. Interestingly, an earlier study on AgamOBP20 concludes that the movement of helices α4 and α5 may be important for activity [27]. In order to ascertain the degree and nature of conformational changes, longer CpHMD simulations, possibly coupled with other computational techniques (e.g. replica exchange), would be necessary.

### Binding sub-sites that can be explored in the design of potential repellents

Ligand binding affinity alone is not a good guide for the design of potential repellents. Mosquito OBPs are promiscuous and can bind a large repertoire of ligand structures some of which are completely non-polar (e.g. α-humulene and β-caryophyllene) [53]. Considering that ligand binding is driven mainly by hydrophobic interactions and that free-energy contributions per heavy atom can be up to 6.2 kJmol^-1^ across a wide variety of macromolecule–small molecule interactions [58], we suggest that it is the binding specificity that determines ligand discrimination rather than binding affinity.

The conserved water molecule observed in the X-ray structures of AgamOBP1 with DEET, Icaridin and 6-MH may thus be an important feature in determining ligand specificity. Water at the interface of a complex is known to provide increased affinity and specificity [59]. Pairwise per-residue decomposition analysis provides a quantitative measure of the contribution of this water molecule to enhancing the binding affinity DEET to AgamOBP1 (S5 Table). The presence of this water molecule, in combination with the structural features of residues lining the central cavity of AgamOBP1 and the adjacent hydrophobic channel, can be explored in synthesising novel repellents. Ligand scaffolds can be designed such that ligands bind with partial occupancies in each of the binding sub-sites of the protein. On the basis of the results reported here, we propose that a possible pharmacophore should include the following features: (i) moieties that form bridged hydrogen bonds involving Cys95 and Trp114 and/or Met55 and His111; (ii) a moiety able to form hydrogen bonds to backbone NH or carbonyl of Phe123 or His111; (iii) an aromatic moiety (e.g., corresponding to tolyl in DEET) that could form stacked π-π interactions with Phe123.

## Supporting information

S1 Table RMS deviation of backbone atoms of AgamOBP1 dimer. (A) First set of MD simulations. (B) Second set of MD simulations.
(DOCX)

S2 Table “Effective” energies of binding. AgamOBP1 dimer apoprotein and in complex with DEET and 6-MH.
(DOCX)

S3 Table “Effective” energies of binding. AgamOBP1 dimer apoprotein and in complex with DEET, 6-MH and Icaridin.
(DOCX)

S4 Table Ligand contributions to the “effective” free energy of binding of the two subunits of AgamOBP1 dimer. Per-residue decomposition of “effective” free energies of binding.
(DOCX)

S5 Table Ligand pairwise per-residue energy decomposition analysis. (A) AgamOBP1 dimer in complex with DEET. (B) AgamOBP1 dimer in complex with 6-MH.
(DOCX)

S6 Table “Effective” energies of binding of DEET and 6-MH to AgamOBP1 dimer. (DOCX)

S7 Table “Effective” energies of binding of Icaridin to AgamOBP1 dimer. (DOCX)

S8 Table Backbone RMS deviation of AgamOBP1 monomers in complex with DEET and 6-MH. (DOCX)

S9 Table “Effective” energies of binding of DEET and 6-MH to AgamOBP1 in multiligand complexes. (DOCX)

S10 Table Pairwise per residue decomposition of “effective energies” of binding. (A) AgamOBP1[chain A]—DEET multiligand complex. (B) AgamOBP1[chain A] - 6MH multiligand complex.
(DOCX)

S11 Table Protonation states of AgamOBP1 residues. (A) Protonation states of ASP, GLU and HIS residues at pH 7.0. (B) Protonation states of ASP, GLU and HIS residues at pH 5.0.
(DOCX)

S1 Fig Principal Components Analysis (PCA). AgamOBP1 dimer in complex with DEET and 6-MH. PC1: Comparison of most variable regions.
(DOCX)

S2 Fig Principal Components Analysis (PCA). **AgamOBP1 dimer in complex with DEET.** PCA scatterplots.
(DOCX)

S3 Fig Principal Components Analysis (PCA). **AgamOBP1 dimer in complex with 6-MH.** PCA scatterplots.
(DOCX)

S4 Fig Secondary structure content (DSSP). AgamOBP1 dimer in complex with DEET (subunit A).
(DOCX)

S5 Fig Secondary structure content (DSSP). AgamOBP1 dimer in complex with 6-MH (subunit A).
(DOCX)

S6 Fig Secondary structure content (DSSP). AgamOBP1 dimer apoprotein (subunit A).
(DOCX)

S7 Fig Secondary structure content (DSSP). AgamOBP1-DEET multiligand complex.
(DOCX)

S8 Fig Secondary structure content (DSSP). AgamOBP1-6MHmultiligand complex.
(DOCX)

S9 Fig AgamOBP1 multiligand complexes (DEET / 6-MH). Principal Component 1: Comparison of most variable regions.
(DOCX)

S10 Fig AgamOBP1-DEET multiligand complex. PCA scatterplots.
(DOCX)

S11 Fig AgamOBP1-6MH multiligand complex. PCA scatterplots.
(DOCX)

S12 Fig CpHMD simulations. **AgamOBP1 monomer.** Time-series of RMSD of backbone atoms of AgamOBP1 from the starting structures over 20 ns of MD simulations.
(DOCX)

S13 Fig CpHMD simulations. RMSF of backbone atoms of subunit A of the AgamOBP1 apoprotein and in complex with Icaridin at pH 7 and pH 5.
(DOCX)

S14 Fig CpHMD simulations. Secondary structure content (DSSP) of AgamOBP1 apoprotein at pH 7 and pH 5.
(DOCX)
