# Supplementary material for: New insights on repellent recognition by Anopheles gambiae odorant-binding protein 1
Source: PLoS One. 2018 Apr 3;13(4):e0194724. doi: 10.1371/journal.pone.0194724 (PMC5882127; doi:10.1371/journal.pone.0194724)
Supplement: S1 Table — (A) First set of MD simulations. (B) Second set of MD simulations. (DOCX) [file pone.0194724.s001.docx]

**S1 Table. RMS deviation of backbone atoms of AgamOBP1 dimer**

**First set of MD simulations**

| **First set of simulations conducted with Amber 12.**  **avg = average rmsd value; σ = standard deviation; Ref structure refers to PDB ID.** |
| --- |
| First set of simulations conducted with Amber 12.  avg = average rmsd value; σ = standard deviation; Ref structure refers to PDB ID. |

| **Ref. structure** | **Chain** | **Ligand** | **Residues** | **RMSD_avg_ (nm)** | **σ (nm)** | **RMSD_min_ (nm)** | **RMSD_max_ (nm)** | **Time (ns)** |
| --- | --- | --- | --- | --- | --- | --- | --- | --- |
| **3N7H** | A | DEET | 1-125 | 0.15 | 0.02 | 0.07 | 0.25 | 100 |
|  | B | DEET | 126-250 | 0.15 | 0.03 | 0.08 | 0.24 | 100 |
| **4FQT** | A | 6-MH | 1-125 | 0.17 | 0.02 | 0.09 | 0.25 | 100 |
|  | B | 6-MH | 126-250 | 0.16 | 0.03 | 0.08 | 0.25 | 100 |
| **4FQT** | A | none | 1-125 | 0.18 | 0.02 | 0.09 | 0.25 | 100 |
|  | B | none | 126-250 | 0.20 | 0.05 | 0.08 | 0.30 | 100 |

**Second set of MD simulations**

| **Ref. structure** | **Chain** | **Ligand** | **Residues** | **RMSD_avg_ (nm)** | **σ (nm)** | **RMSD_min_ (nm)** | **RMSD_max_ (nm)** | **Time (ns)** |
| --- | --- | --- | --- | --- | --- | --- | --- | --- |
| **3N7H** | A | DEET | 11-125 | 0.14 | 0.02 | 0.07 | 0.23 | 100 |
|  | B | DEET | 126-250 | 0.10 | 0.01 | 0.05 | 0.18 | 100 |
| **4FQT** | A | 6-MH | 1-125 | 0.13 | 0.01 | 0.07 | 0.20 | 100 |
|  | B | 6-MH | 126-250 | 0.11 | 0.01 | 0.05 | 0.17 | 100 |
| **4FQT** | A | none | 1-125 | 0.16 | 0.02 | 0.10 | 0.24 | 100 |
|  | B | none | 126-250 | 0.14 | 0.04 | 0.05 | 0.30 | 100 |

MD simulations conducted with Amber 16. **avg** = average rmsd value; **σ** = standard deviation; **Ref. structure** refers to PDB ID.
