# Supplementary material for: New insights on repellent recognition by Anopheles gambiae odorant-binding protein 1
Source: PLoS One. 2018 Apr 3;13(4):e0194724. doi: 10.1371/journal.pone.0194724 (PMC5882127; doi:10.1371/journal.pone.0194724)
Supplement: S2 Table — AgamOBP1 dimer apoprotein and in complex with DEET and 6-MH. (DOCX) [file pone.0194724.s002.docx]

# S2 Table. “Effective” energies of binding

# AgamOBP1 dimer apoprotein and in complex with DEET and 6-MH

|  | ***Agam*OBP1_[A:B]_** | | ***Agam*OBP1_[A:B]_** | | ***Agam*OBP1_[A:B]_** | |
| --- | --- | --- | --- | --- | --- | --- |
| **Ligand** | **DEET** | | **6-MH** | | **none** | |
| Contrib.^a^ | Δ value ^b^ | σ^c^ | Δ value ^b^ | σ^c^ | Δ value ^b^ | σ^c^ |
| *ΔH_vdW_* | -339.7 | 29.7 | -274.5 | 24.6 | -241.1 | 25.3 |
| *ΔH_elec_* | 2175.3 | 198.4 | 2108.2 | 221.1 | 2581.9 | 279.5 |
| ***ΔH_gas_*** | 1835.6 | 201.9 | 1833.7 | 229.5 | 2340.8 | 278.3 |
| *ΔG_GB_* | -1994.8 | 182.7 | -1951.5 | 212.0 | -2423.7 | 266.6 |
| *ΔG_np_* | -45.2 | 3.2 | -39.4 | 3.3 | -33.1 | 3.1 |
| ***ΔG_solv_*** | -2040.1 | 181.4 | -1990.9 | 209.8 | -2456.9 | 265.8 |
| ***ΔG_gas+solv_*** | -204.4 | 34.7 | -157.2 | 30.1 | -116.1 | 24.4 |

***^a^*** *ΔH_elec,_=Coulombic energy; ΔH_vdW_ =van der Waals energy; ΔG_GB_ =polar solvation free energy; ΔG_np_ =non-polar solvation free energy; ΔH_gas_ = ΔH_elec_ + ΔH_vdW;_ ΔG_solv_ = ΔG_GB_ + ΔG_np_; ΔG_gas+solv_ = ΔH_gas_ + ΔG_solv_*

**^b^** Average difference (Complex - Receptor - Ligand); **^c^** Standard deviation. Energy values in kJ mol^-1^
