# Supplementary material for: New insights on repellent recognition by Anopheles gambiae odorant-binding protein 1
Source: PLoS One. 2018 Apr 3;13(4):e0194724. doi: 10.1371/journal.pone.0194724 (PMC5882127; doi:10.1371/journal.pone.0194724)
Supplement: S3 Table — AgamOBP1 dimer apoprotein and in complex with DEET, 6-MH and Icaridin. (DOCX) [file pone.0194724.s003.docx]

# S3 Table. “Effective” energies of binding

# AgamOBP1 dimer apoprotein and in complex with DEET, 6-MH and Icaridin

| **Ligand** | **DEET** | | **Icaridin** | | **6-MH** | | **none** | |
| --- | --- | --- | --- | --- | --- | --- | --- | --- |
| Contrib.^a^ | Δ value ^b^ | σ^c^ | Δ value ^b^ | σ^c^ | Δ value ^b^ | σ^c^ | Δ value ^b^ | σ^c^ |
| *ΔH_vdW_* | -294.4 | 30.5 | -263.4 | 20.6 | -168.3 | 24.8 | -213.2 | 21.8 |
| *ΔH_elec_* | 1645.8 | 208.0 | 1671.3 | 188.8 | 1674.3 | 158.8 | 1790.5 | 255.3 |
| ***ΔH_gas_*** | 1351.4 | 212.2 | 1408.0 | 188.1 | 1441.7 | 164.2 | 1577.3 | 227.3 |
| *ΔG_GB_* | -1544.3 | 196.7 | -1161.8 | 177.5 | -1610.1 | 157.0 | -1724.8 | 211.9 |
| *ΔG_np_* | -45.3 | 4.3 | -40.9 | 1.9 | -36.4 | 2.95 | -33.6 | 2.6 |
| ***ΔG_solv_*** | -1589.6 | 195.0 | -1647.3 | 176.7 | -1646.5 | 155.8 | -1578.4 | 210.7 |
| ***ΔG_gas+solv_*** | -238.2 | 31.3 | -239.4 | 23.0 | -204.8 | 20.3 | -181.1 | 28.2 |

***^a^*** *ΔH_elec,_=Coulombic energy; ΔH_vdW_ =van der Waals energy; ΔG_GB_ =polar solvation free energy; ΔG_np_ =non-polar solvation free energy; ΔH_gas_ = ΔH_elec_ + ΔH_vdW;_ ΔG_solv_ = ΔG_GB_ + ΔG_np_; ΔG_gas+solv_ = ΔH_gas_ + ΔG_solv_*

**^b^** Average difference (Complex - Receptor - Ligand); **^c^** Standard deviation. Energy values in kJ mol^-1^

**MMGBA calculations from repeat MD simulations of AgamOBP1 DEET, 6-MH complexes and AgamOBP1 apoprotein (cf. S8 Table)**
