# Supplementary material for: New insights on repellent recognition by Anopheles gambiae odorant-binding protein 1
Source: PLoS One. 2018 Apr 3;13(4):e0194724. doi: 10.1371/journal.pone.0194724 (PMC5882127; doi:10.1371/journal.pone.0194724)

**S4 Table. Ligand contributions to the “effective” free energy of binding of the two subunits of AgamOBP1 dimer.**

**Per-residue decomposition of “effective” free energies of binding**

Residues making the greatest contribution towards the “effective”energy (ΔGgas+solv) of binding of chains A and B of AgamOBP1 in the presence an d absence of ligand. Units in kJ/mol. The contribution of Icaridin towards the “effective” energy of binding of chains A and B was found to be -34.0 kJ/mol

| **Residue No.** | **AgamOBP1-DEET** | **AgamOBP1-6MH** | **AgamOBP1 apoprotein** | |
| --- | --- | --- | --- | --- |
| D66 | 4.9 | 5.4 | 3.7 |  |
| D70 | 7.5 | 5.6 | 5.1 |  |
| H72 | -10.0 | -9.8 | -1.0 |  |
| L73 | -19.5 | -16.8 | -18.9 |  |
| E74 | 18.5 | 15.3 | 3.6 |  |
| H77 | -14.3 | -19.0 | -15.7 |  |
| D86 | 12.5 | 12.1 | 2.1 |  |
| M89 | -22.3 | -21.0 | -25.8 |  |
| K93 | -13.2 | -12.4 | -24.4 |  |
| R94 | -45.6 | -51.6 | -17.3 |  |
| C95 | -10.9 | -3.5 | 0.2 |  |
| L96 | -43.4 | -37.7 | -28.8 |  |
| Y97 | -24.1 | -25.5 | -17.7 |  |
| E99 | 6.1 | 6.5 | 7.4 |  |
| W114 | -15.1 | -0.6 | 0.2 |  |
| D118 | 3.4 | 3.3 | 3.5 |  |
| LIG | -30.4 | -17.4 | 0.0 |  |


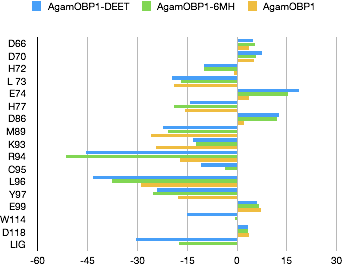

Supplement: S4 Table — Per-residue decomposition of “effective” free energies of binding. (DOCX) [file pone.0194724.s004.docx]
