# Supplementary material for: New insights on repellent recognition by Anopheles gambiae odorant-binding protein 1
Source: PLoS One. 2018 Apr 3;13(4):e0194724. doi: 10.1371/journal.pone.0194724 (PMC5882127; doi:10.1371/journal.pone.0194724)
Supplement: S5 Table — (A) AgamOBP1 dimer in complex with DEET. (B) AgamOBP1 dimer in complex with 6-MH. (DOCX) [file pone.0194724.s005.docx]

**S5 Table. Ligand pairwise per-residue energy decomposition analysis**

| 1. **AgamOBP1 dimer in complex with DEET** | | | | | | |
| --- | --- | --- | --- | --- | --- | --- |
| **Residue1** | **Residue2** | **vdWaals** | **Electrostatic** | **Polar Solv.** | **Non-polar solv** | **Total** |
| DEET[B] | HOH | 2.1 | -16.8 | 4.2 | -1.5 | -12.0 |
| DEET[A] | HOH | 2.2 | -16.1 | 3.5 | -1.4 | -11.9 |
| DEET[A] | DEET[B] | -3.2 | 0.2 | 0.1 | -3.2 | -6.2 |
| DEET[B] | LEU 96[A] | -3.3 | 0.5 | -0.4 | -2.9 | -6.0 |
| DEET[A] | LEU 96[B] | -3.0 | 0.5 | -0.4 | -2.6 | -5.5 |
| DEET[B] | LYS 93[A] | -2.0 | 3.4 | -3.4 | -1.4 | -3.4 |
| DEET[A] | LYS 93[B] | -1.7 | 3.6 | -3.6 | -1.3 | -3.0 |
| DEET[B] | MET 89[A] | -1.5 | -0.8 | 0.9 | -0.9 | -2.3 |
| DEET[A] | MET 215[B] | -1.1 | -0.5 | 0.6 | -0.5 | -1.5 |

Generalised Born solvent. Units in kJ/mol

| 1. **AgamOBP1 dimer in complex with 6-MH** | | | | | | |
| --- | --- | --- | --- | --- | --- | --- |
| **Residue1** | **Residue2** | **vdWaals** | **Electrostatic** | **Polar Solv.** | **Non-polar solv** | **Total** |
| 6-MH[A] | HOH | -2.5 | 0.0 | 0.1 | -2.7 | -5.1 |
| 6-MH[A] | HOH | -2.5 | -0.7 | 0.7 | -2.1 | -4.7 |
| 6-MH[B] | DEET[B] | -1.7 | 0.1 | 0.0 | -1.7 | -3.3 |
| 6-MH[A] | LEU 96[A] | -1.5 | -0.2 | 0.2 | -1.3 | -2.7 |
| 6-MH[B] | LEU 96[B] | -1.4 | 0.3 | -0.3 | -1.2 | -2.6 |
| 6-MH[B] | LYS 93[A] | -1.3 | 0.1 | 0.0 | -1.4 | -2.6 |
| 6-MH[B] | LYS 93[B] | -0.8 | -0.0 | 0.2 | -0.5 | -1.3 |
| 6-MH[B] | MET 89[A] | -0.2 | -0.2 | 0.1 | -0.2 | -0.5 |

Generalised Born solvent. Units in kJ/mol
