# Supplementary material for: New insights on repellent recognition by Anopheles gambiae odorant-binding protein 1
Source: PLoS One. 2018 Apr 3;13(4):e0194724. doi: 10.1371/journal.pone.0194724 (PMC5882127; doi:10.1371/journal.pone.0194724)
Supplement: S6 Table — (DOCX) [file pone.0194724.s006.docx]

**S6 Table. “Effective” energies of binding of DEET and 6-MH to AgamOBP1 dimer**

| ***1^st^ set of simulations*** | | | | | | | | |
| --- | --- | --- | --- | --- | --- | --- | --- | --- |
|  | *DEET_sub_*_A_ | | *DEET_subB_* | | *6-MH_sub_*_A_ | | *6-MH_subB_* | |
| Contrib.^a^ | Δ value ^b^ | σ^c^ | Δ value ^b^ | σ^c^ | Δ value ^b^ | σ^c^ | Δ value ^b^ | σ^c^ |
| *ΔH_vdW_* | -146.1 | 9.6 | -146.6 | 8.8 | -108.6 | 6.8 | -101.0 | 10.4 |
| *ΔH_elec_* | -38.1 | 9.4 | -35.1 | 8.2 | -14.2 | 7.3 | -11.5 | 9.9 |
| ***ΔH_gas_*** | -184.3 | 10.8 | -181.7 | 9.2 | -122.8 | 10.0 | -112.5 | 15.3 |
| *ΔG_GB_* | 69.4 | 6.0 | 65.4 | 5.7 | 44.8 | 5.9 | 42.2 | 9.0 |
| *ΔG_np_* | -19.5 | 0.4 | -19.6 | 0.4 | -15.2 | 0.4 | -14.6 | 1.1 |
| ***ΔG_solv_*** | 49.9 | 6.0 | 45.8 | 5.7 | 29.6 | 5.9 | 27.6 | 8.7 |
| ***ΔG_gas+sol_*** | -134.4 | 8.9 | -135.9 | 8.3 | -93.2 | 7.5 | -85.0 | 10.8 |

| ***2^nd^ set of simulations*** | | | | | | | | |
| --- | --- | --- | --- | --- | --- | --- | --- | --- |
|  | *DEET_sub_*_A_ | | *DEET_subB_* | | *6-MH_sub_*_A_ | | *6-MH_subB_* | |
| Contrib.^a^ | Δ value ^b^ | σ^c^ | Δ value ^b^ | σ^c^ | Δ value ^b^ | σ^c^ | Δ value ^b^ | σ^c^ |
| *ΔH_vdW_* | -145.5 | 8.4 | -147.8 | 8.8 | -88.8 | 9.3 | -110.0 | 6.9 |
| *ΔH_elec_* | -32.5 | 9.7 | -31.9 | 9.7 | -8.0 | 7.2 | -15.9 | 7.9 |
| ***ΔH_gas_*** | -178.0 | 10.7 | -179.7 | 10.5 | -96.0 | 13.2 | -125.9 | 10.3 |
| *ΔG_GB_* | 69.0 | 5.8 | 64.0 | 5.6 | 36.7 | 7.8 | 45.6 | 6.2 |
| *ΔG_np_* | -19.4 | 0.4 | -19.2 | 0.4 | -13.1 | 0.9 | -15.4 | 0.5 |
| ***ΔG_solv_*** | 49.6 | 5.8 | 44.8 | 5.7 | 25.6 | 7.4 | 30.2 | 6.1 |
| ***ΔG_gas+sol_*** | -128.4 | 8.6 | -134.9 | 8.5 | -71.2 | 9.3 | -95.6 | 7.6 |

_subA_ and _subB_ refer to protein subunits A and B, respectively.

***^a^*** *ΔH_elec,_=Coulombic energy; ΔH_vdW_ =van der Waals energy; ΔG_GB_ =polar solvation free energy; ΔG_np_ =non-polar solvation free energy; ΔH_gas_ = ΔH_elec_ + ΔH_vdW;_ ΔG_solv_ = ΔG_GB_ + ΔG_np_; ΔG_gas+solv_ = ΔH_gas_ + ΔG_solv_*

**^b^** Average difference (Complex - Receptor - Ligand); **^c^** Standard deviation. Energy values in kJ mol^-1^
