# Supplementary material for: New insights on repellent recognition by Anopheles gambiae odorant-binding protein 1
Source: PLoS One. 2018 Apr 3;13(4):e0194724. doi: 10.1371/journal.pone.0194724 (PMC5882127; doi:10.1371/journal.pone.0194724)
Supplement: S7 Table — (DOCX) [file pone.0194724.s007.docx]

**S7 Table. “Effective” energies of binding of Icaridin to AgamOBP1 dimer**

|  | *Icaridin_sub_*_A_ | | *Icaridin_subB_* | |
| --- | --- | --- | --- | --- |
| Contrib.^a^ | Δ value ^b^ | σ^c^ | Δ value ^b^ | σ^c^ |
| *ΔH_vdW_* | -171.4 | 10.0 | -174.2 | 9.4 |
| *ΔH_elec_* | -63.5 | 11.8 | -46.5 | 19.0 |
| ***ΔH_gas_*** | -235.0 | 12.3 | -220.7 | 20.6 |
| *ΔG_GB_* | 87.4 | 6.2 | 88.6 | 14.0 |
| *ΔG_np_* | -23.0 | 0.4 | -22.9 | 0.5 |
| ***ΔG_solv_*** | 64.4 | 6.1 | 65.9 | 13.7 |
| ***ΔG_gas+sol_*** | -170.5 | 9.4 | -154.9 | 11.4 |

_subA_ and _subB_ refer to protein subunits A and B, respectively.

***^a^*** *ΔH_elec,_=Coulombic energy; ΔH_vdW_ =van der Waals energy; ΔG_GB_ =polar solvation free energy; ΔG_np_ =non-polar solvation free energy; ΔH_gas_ = ΔH_elec_ + ΔH_vdW;_ ΔG_solv_ = ΔG_GB_ + ΔG_np_; ΔG_gas+solv_ = ΔH_gas_ + ΔG_solv_*

**^b^** Average difference (Complex - Receptor - Ligand); **^c^** Standard deviation. Energy values in kJ mol^-1^
