# Supplementary material for: New insights on repellent recognition by Anopheles gambiae odorant-binding protein 1
Source: PLoS One. 2018 Apr 3;13(4):e0194724. doi: 10.1371/journal.pone.0194724 (PMC5882127; doi:10.1371/journal.pone.0194724)
Supplement: S8 Table — (DOCX) [file pone.0194724.s008.docx]

**S8 Table. Backbone RMS deviation of AgamOBP1 monomers in complex with DEET and 6-MH**

| **Ref. structure** | **Ligand** | **Res. No** | **RMSD_avg_ (nm)** | **σ (nm)** | **RMSD_min_ (nm)** | **RMSD_max_ (nm)** | **Time (ns)** |
| --- | --- | --- | --- | --- | --- | --- | --- |
| 3N7H | DEET | 1-125 | 0.16 | 0.02 | 0.09 | 0.23 | 100 |
| 4FQT | 6-MH | 1-125 | 0.17 | 0.02 | 0.09 | 0.25 | 100 |

avg = average rmsd value; σ = standard deviation; Ref structure refers to PDB ID.
