# Supplementary material for: New insights on repellent recognition by Anopheles gambiae odorant-binding protein 1
Source: PLoS One. 2018 Apr 3;13(4):e0194724. doi: 10.1371/journal.pone.0194724 (PMC5882127; doi:10.1371/journal.pone.0194724)
Supplement: S9 Table — (DOCX) [file pone.0194724.s009.docx]

**S9 Table. “Effective” energies of binding of DEET and 6-MH to AgamOBP1 in multiligand complexes**

| **Ligand** | *DEET_X-ray_* | | *DEET_docked_* | | *6-MH_X-ray_* | | *6-MH_docked_* | |
| --- | --- | --- | --- | --- | --- | --- | --- | --- |
| Contrib.^a^ | Δ value ^b^ | σ^c^ | Δ value ^b^ | σ^c^ | Δ value ^b^ | σ^c^ | Δ value ^b^ | σ^c^ |
| *ΔH_vdW_* | -126.0 | 9.8 | -118.9 | 8.8 | -102.8 | 10.3 | -86.2 | 7.6 |
| *ΔH_elec_* | -57.7 | 15.0 | 2.6 | 12.1 | -7.2 | 9.9 | -5.7 | 14.4 |
| ***ΔH_gas_*** | -183.7 | 18.8 | -116.3 | 13.0 | -110.0 | 12.1 | -91.8 | 18.3 |
| *ΔG_GB_* | 88.7 | 11.6 | 42.5 | 11.9 | 39.9 | 9.6 | 32.7 | 14.2 |
| *ΔG_np_* | -17.0 | 0.6 | -16.8 | 0.9 | -14.4 | 1.0 | -13.2 | 1.0 |
| ***ΔG_solv_*** | 71.6 | 11.4 | 25.8 | 11.9 | 25.5 | 9.8 | 19.5 | 13.7 |
| ***ΔG_gas+solv_*** | -112.1 | 11.0 | -90.5 | 9.1 | -84.5 | 11.2 | -72.3 | 8.2 |

Ligand subscripts _X-ray_ and _docked_ refer to ligands derived from the X-ray and docking models, respectively.

***^a^*** *ΔH_elec,_=Coulombic energy; ΔH_vdW_ =van der Waals energy; ΔG_GB_ =polar solvation free energy; ΔG_np_ =non-polar solvation free energy; ΔH_gas_ = ΔH_elec_ + ΔH_vdW;_ ΔG_solv_ = ΔG_GB_ + ΔG_np_; ΔG_gas+solv_ = ΔH_gas_ + ΔG_solv_*

**^b^** Average difference (Complex - Receptor - Ligand); **^c^** Standard deviation. Energy values in kJ mol^-1^
