# Supplementary material for: New insights on repellent recognition by Anopheles gambiae odorant-binding protein 1
Source: PLoS One. 2018 Apr 3;13(4):e0194724. doi: 10.1371/journal.pone.0194724 (PMC5882127; doi:10.1371/journal.pone.0194724)
Supplement: S10 Table — (A) AgamOBP1[chain A]—DEET multiligand complex. (B) AgamOBP1[chain A] - 6MH multiligand complex. (DOCX) [file pone.0194724.s010.docx]

**S10 Table. Pairwise per residue decomposition of “effective energies” of binding.**

**(A) AgamOBP1[chain A] - DEET multiligand complex**

| **Ligand** | **Residue** | **Residue position** | **van der Waals** | **Electrostatic** | **Polar Solvation** | **Non-Polar Solvation** | **Total** |
| --- | --- | --- | --- | --- | --- | --- | --- |
| **DEET[X-ray]** | ALA 88 | helix 5 (DI) | -7.83 | 0.93 | -1.52 | -5.52 | -13.93 |
| **DEET[X-ray]** | TRP 114 | helix 6 (DI) | -5.41 | -3.67 | 3.96 | -3.92 | -9.05 |
| **DEET[X-ray]** | LEU 76 | helix 4 (DI) | -4.37 | 0.23 | 0.04 | -3.26 | -7.36 |
| **DEET[X-ray]** | MET 91 | helix 5 (DI) | -3.52 | 1.24 | -1.98 | -2.49 | -6.76 |
| **DEET[X-ray]** | LEU 80 | loop (helix 4-5, DI) | -2.21 | 0.24 | -0.21 | -2.00 | -4.19 |
| **DEET[X-ray]** | DEET[docked] |  | -1.94 | -0.07 | 0.39 | -1.87 | -3.49 |
|  |  |  |  |  |  |  |  |
| **DEET[docked]** | PHE 123 | C-terminous | -6.94 | -0.68 | 1.57 | -5.63 | -11.68 |
| **DEET[docked]** | LEU 15 | helix 1 | -4.93 | 0.11 | -0.18 | -4.23 | -9.23 |
| **DEET[docked]** | LEU 76 | helix 4 | -4.60 | 1.18 | -1.04 | -3.98 | -8.44 |
| **DEET[docked]** | LEU 19 | helix 1 | -4.48 | -0.53 | 0.58 | -3.81 | -8.24 |
| **DEET[docked]** | LEU 80 | loop (helix 4-5, DI) | -3.55 | -0.43 | 0.30 | -2.83 | -6.50 |
| **DEET[docked]** | TRP 114 | helix 6 (DI) | -3.65 | -0.64 | 1.17 | -2.91 | -6.02 |
| **DEET[docked]** | LEU 58 | helix 3 | -2.91 | -0.66 | 0.46 | -2.26 | -5.37 |
| **DEET[docked]** | PHE 59 | helix 3 | -2.82 | 0.02 | 0.28 | -2.06 | -4.58 |

Energy in kJ/mol; DI = dimer interface

**S21. Pairwise per residue decomposition of “effective energies” of binding.**

**(B) AgamOBP1[chain A] - 6MH multiligand complex**

| **Ligand** | **Residue** | **Residue position** | **van der Waals** | **Electrostatic** | **Polar Solvation** | **Non-Polar Solvation** | **TOTAL** |
| --- | --- | --- | --- | --- | --- | --- | --- |
| **6-MH[X-ray]** | TRP 114 | helix 6 (DI) | -8.29 | -1.04 | 0.53 | -6.58 | -15.39 |
| **6-MH[X-ray]** | MET 91 | helix 5 (DI) | -4.28 | -0.66 | 0.66 | -3.49 | -7.77 |
| **6-MH[X-ray]** | PHE 123 | C-terminous | -3.44 | -0.49 | 0.68 | -3.14 | -6.38 |
| **6-MH[X-ray]** | ALA 88 | helix 5 (DI) | -3.30 | -1.34 | 1.01 | -2.65 | -6.29 |
| **6-MH[X-ray]** | TYR 122 | C-terminous | -3.36 | 0.42 | -0.66 | -2.35 | -5.94 |
| **6-MH[X-ray]** | LEU 80 | loop (helix 4-5, DI) | -2.78 | -0.34 | 0.30 | -2.72 | -5.54 |
| **6-MH[X-ray]** | ILE 87 | helix 5 (DI) | -2.85 | -1.12 | 0.43 | -1.93 | -5.48 |
| **6-MH[X-ray]** | MET 84 | helix 5 (DI) | -2.60 | 0.36 | -0.30 | -2.29 | -4.84 |
| **6-MH[X-ray]** | LEU 76 | helix 4 (DI) | -2.55 | 0.26 | -0.51 | -1.97 | -4.77 |
| **6-MH[X-ray]** | 6-MH[docked] | NA | -2.28 | -0.05 | 0.05 | -2.35 | -4.63 |
|  |  |  |  |  |  |  |  |
| **6-MH[docked]** | LEU 58 | helix 3 | -3.52 | -0.76 | 0.49 | -2.85 | -6.64 |
| **6-MH[docked]** | LEU 19 | helix 1 | -3.49 | 0.16 | -0.17 | -3.14 | -6.64 |
| **6-MH[docked]** | LEU 76 | helix 4 | -3.42 | 0.12 | -0.04 | -2.92 | -6.26 |
| **6-MH[docked]** | LEU 15 | helix 1 | -2.97 | -0.26 | 0.22 | -2.89 | -5.90 |
| **6-MH[docked]** | LEU 80 | loop (helix 4-5, DI) | -2.84 | 0.33 | -0.28 | -2.38 | -5.18 |
| **6-MH[docked]** | SER 79 | helix 4 | -2.34 | -0.74 | 0.06 | -2.14 | -5.16 |
| **6-MH[docked]** | PHE 59 | helix 3 | -3.06 | 0.07 | 0.38 | -2.43 | -5.04 |
| **6-MH[docked]** | ALA 62 | loop (helix 2-3) | -2.39 | 0.57 | -0.57 | -2.49 | -4.89 |
| **6-MH[docked]** | PHE 123 | C-terminous | -2.00 | -0.72 | 0.22 | -1.82 | -4.32 |

Energy in kJ/mol; DI = dimer interface
