# Supplementary material for: New insights on repellent recognition by Anopheles gambiae odorant-binding protein 1
Source: PLoS One. 2018 Apr 3;13(4):e0194724. doi: 10.1371/journal.pone.0194724 (PMC5882127; doi:10.1371/journal.pone.0194724)
Supplement: S11 Table — (A) Protonation states of ASP, GLU and HIS residues at pH 7.0. (B) Protonation states of ASP, GLU and HIS residues at pH 5.0. (DOCX) [file pone.0194724.s011.docx]

# S11 Table. Protonation states of AgamOBP1 residues

1. **Protonation states ASP, GLU and HIS residues at pH 7.0**

| **Res** | **Position** | **Offset** | **Pred** | **FracProt** | **Transitions** |
| --- | --- | --- | --- | --- | --- |
| ASP | 1 | -4.301 | 2.699 | 0 | 6 |
| ASP | 7 | -inf | -inf | 0 | 0 |
| GLU | 9 | -1.706 | 5.294 | 0.019 | 734 |
| GLU | 14 | -1.816 | 5.184 | 0.015 | 766 |
| GLU | 17 | -1.717 | 5.283 | 0.019 | 858 |
| HIS | 23 | 1.583 | 8.583 | 0.975 | 501 |
| ASP | 24 | -2.931 | 4.069 | 0.001 | 56 |
| GLU | 34 | -1.699 | 5.301 | 0.02 | 171 |
| GLU | 35 | -2.667 | 4.333 | 0.002 | 86 |
| ASP | 42 | -2.25 | 4.75 | 0.006 | 266 |
| GLU | 43 | -0.88 | 6.12 | 0.116 | 846 |
| GLU | 44 | -2.737 | 4.263 | 0.002 | 92 |
| HIS | 46 | 0.088 | 7.088 | 0.55 | 16607 |
| GLU | 47 | -1.654 | 5.346 | 0.022 | 558 |
| ASP | 48 | -3.252 | 3.748 | 0.001 | 24 |
| GLU | 49 | -2.189 | 4.811 | 0.006 | 278 |
| HIS | 60 | -1.262 | 5.738 | 0.052 | 3903 |
| GLU | 61 | -1.927 | 5.073 | 0.012 | 542 |
| ASP | 66 | -2.82 | 4.18 | 0.002 | 74 |
| ASP | 67 | -2.766 | 4.234 | 0.002 | 70 |
| ASP | 70 | -1.135 | 5.865 | 0.068 | 1412 |
| HIS | 72 | -1.384 | 5.616 | 0.04 | 2648 |
| GLU | 74 | -1.984 | 5.016 | 0.01 | 420 |
| HIS | 77 | -0.721 | 6.279 | 0.16 | 5421 |
| ASP | 78 | -2.358 | 4.642 | 0.004 | 218 |
| HIS | 85 | 0.471 | 7.471 | 0.748 | 1825 |
| ASP | 86 | -3.017 | 3.983 | 0.001 | 44 |
| HIS | 90 | -0.855 | 6.145 | 0.123 | 7296 |
| GLU | 99 | -1.069 | 5.931 | 0.079 | 2064 |
| GLU | 101 | -1.824 | 5.176 | 0.015 | 682 |
| ASP | 105 | -4.046 | 2.954 | 0 | 4 |
| HIS | 111 | -0.289 | 6.711 | 0.339 | 3881 |
| ASP | 118 | -2.219 | 4.781 | 0.006 | 134 |
| HIS | 121 | -0.474 | 6.526 | 0.251 | 7313 |
| **Offset:** is is the difference between the predicted pK_a_ and the system pH; **Pred:** is is the predicted pK_a_; **FracProt:** is the fraction of time the residue spends protonated; **%:** is the occupancy of hydrogen bonds. **Transitions*:*** gives the number of accpeted protonations state transitions | | | | | |

**(B) Protonation states ASP, GLU and HIS residues at pH 5.0**

| **Res** | **Position** | **Offset** | **Pred** | **FracProt** | **Transitions** |
| --- | --- | --- | --- | --- | --- |
| ASP | 1 | -1.461 | 3.539 | 0.033 | 4354 |
| ASP | 7 | -1.041 | 3.959 | 0.083 | 6294 |
| GLU | 9 | -0.147 | 4.853 | 0.416 | 31026 |
| GLU | 14 | 0.056 | 5.056 | 0.532 | 37290 |
| GLU | 17 | -0.382 | 4.618 | 0.293 | 28272 |
| HIS | 23 | 1.944 | 6.944 | 0.989 | 518 |
| ASP | 24 | -1.242 | 3.758 | 0.054 | 10046 |
| GLU | 34 | -1.047 | 3.953 | 0.082 | 7452 |
| GLU | 35 | -1.044 | 3.956 | 0.083 | 13204 |
| ASP | 42 | -1.334 | 3.666 | 0.044 | 4802 |
| GLU | 43 | 0.835 | 5.835 | 0.872 | 7205 |
| GLU | 44 | -0.479 | 4.521 | 0.249 | 14063 |
| HIS | 46 | 1.133 | 6.133 | 0.931 | 19658 |
| GLU | 47 | -0.346 | 4.654 | 0.311 | 13765 |
| ASP | 48 | -0.882 | 4.118 | 0.116 | 5588 |
| GLU | 49 | -0.369 | 4.631 | 0.299 | 25254 |
| HIS | 60 | 0.463 | 5.463 | 0.744 | 37254 |
| GLU | 61 | -1.021 | 3.979 | 0.087 | 8870 |
| ASP | 66 | -1.815 | 3.185 | 0.015 | 2158 |
| ASP | 67 | -1.167 | 3.833 | 0.064 | 9572 |
| ASP | 70 | -0.56 | 4.44 | 0.216 | 21144 |
| HIS | 72 | 0.932 | 5.932 | 0.895 | 15213 |
| GLU | 74 | -0.737 | 4.263 | 0.155 | 21742 |
| HIS | 77 | 0.427 | 5.427 | 0.728 | 43622 |
| ASP | 78 | -1.107 | 3.893 | 0.072 | 11918 |
| HIS | 85 | 1.003 | 6.003 | 0.91 | 13468 |
| ASP | 86 | -1.789 | 3.211 | 0.016 | 2864 |
| HIS | 90 | 0.538 | 5.538 | 0.775 | 26144 |
| GLU | 99 | 0.417 | 5.417 | 0.723 | 37689 |
| GLU | 101 | -0.184 | 4.816 | 0.396 | 41929 |
| ASP | 105 | -2.648 | 2.352 | 0.002 | 356 |
| HIS | 111 | 1.828 | 6.828 | 0.985 | 2772 |
| ASP | 118 | -2.321 | 2.679 | 0.005 | 118 |
| **Offset:** is is the difference between the predicted pK_a_ and the system pH; **Pred:** is is the predicted pK_a_; **FracProt:** is the fraction of time the residue spends protonated; **%:** is the occupancy of hydrogen bonds. **Transitions*:*** gives the number of accpeted protonations state transitions | | | | | |
