# Supplementary material for: New insights on repellent recognition by Anopheles gambiae odorant-binding protein 1
Source: PLoS One. 2018 Apr 3;13(4):e0194724. doi: 10.1371/journal.pone.0194724 (PMC5882127; doi:10.1371/journal.pone.0194724)
Supplement: S1 Fig — AgamOBP1 dimer in complex with DEET and 6-MH. PC1: Comparison of most variable regions. (DOCX) [file pone.0194724.s012.docx]

# S1 Fig. Principal Components Analysis (PCA)


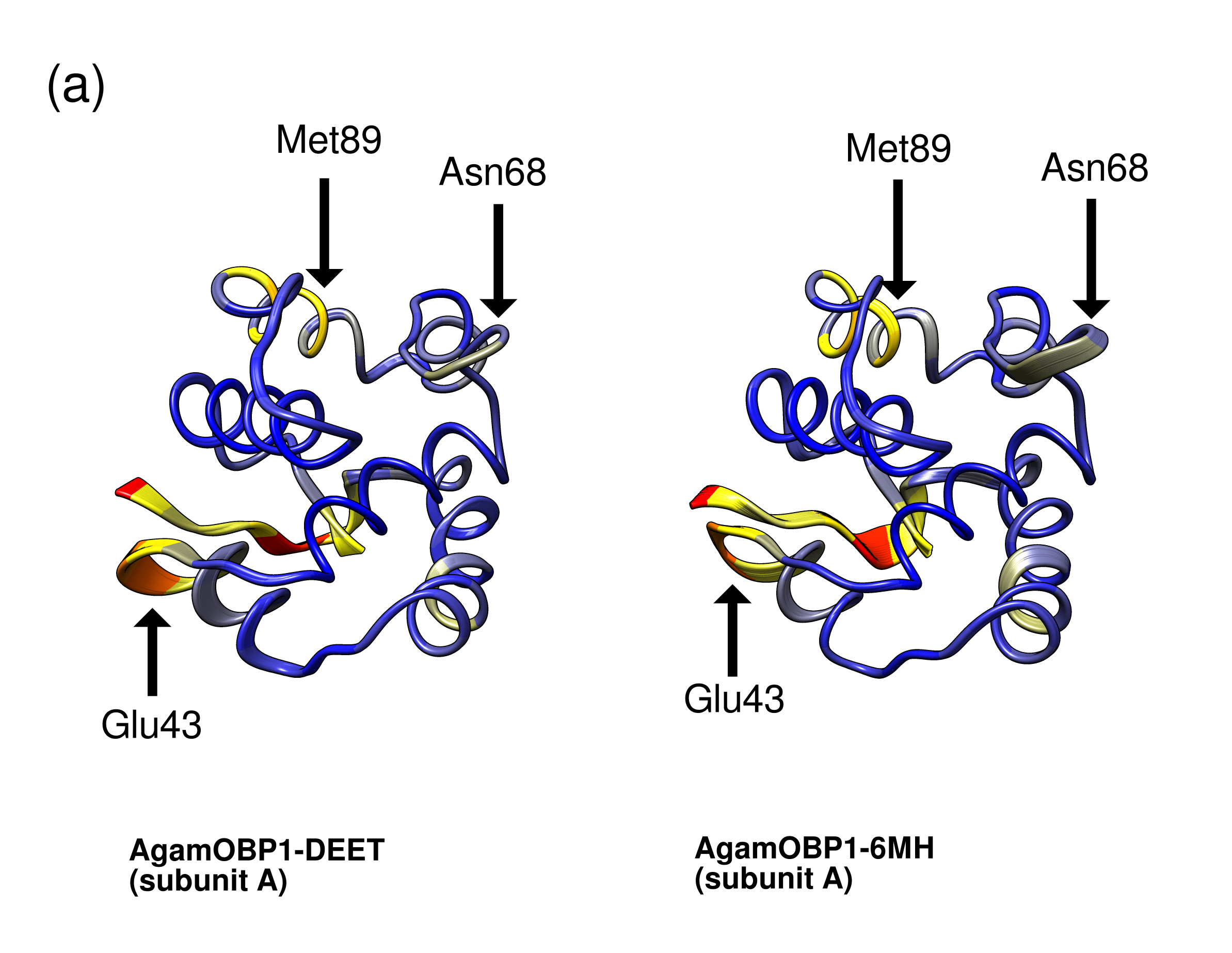


# AgamOBP1 dimer in complex with DEET and 6-MH. Principal Component 1: Comparison of most variable regions
