# Supplementary figures and images for: New insights on repellent recognition by Anopheles gambiae odorant-binding protein 1
Source: PLoS One. 2018 Apr 3;13(4):e0194724. doi: 10.1371/journal.pone.0194724 (PMC5882127; doi:10.1371/journal.pone.0194724)

# S2 Fig. Principal Components Analysis (PCA)

# AgamOBP1 dimer in complex with DEET

**PCA scatterplots**


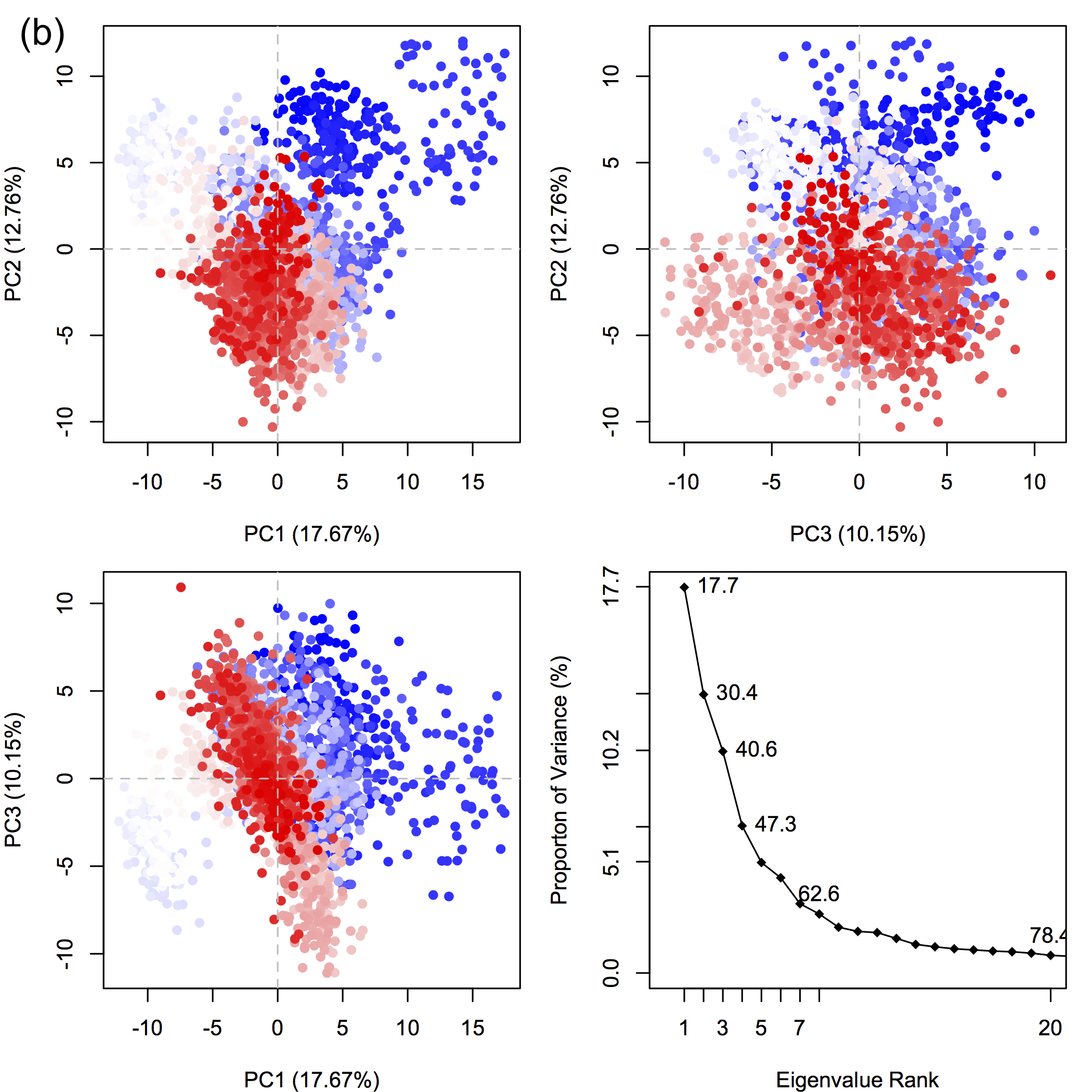

Supplement: S2 Fig — AgamOBP1 dimer in complex with DEET. PCA scatterplots. (DOCX) [file pone.0194724.s013.docx]

# S3 Fig. Principal Components Analysis (PCA)

# AgamOBP1 dimer in complex with 6-MH

**PCA scatterplots**


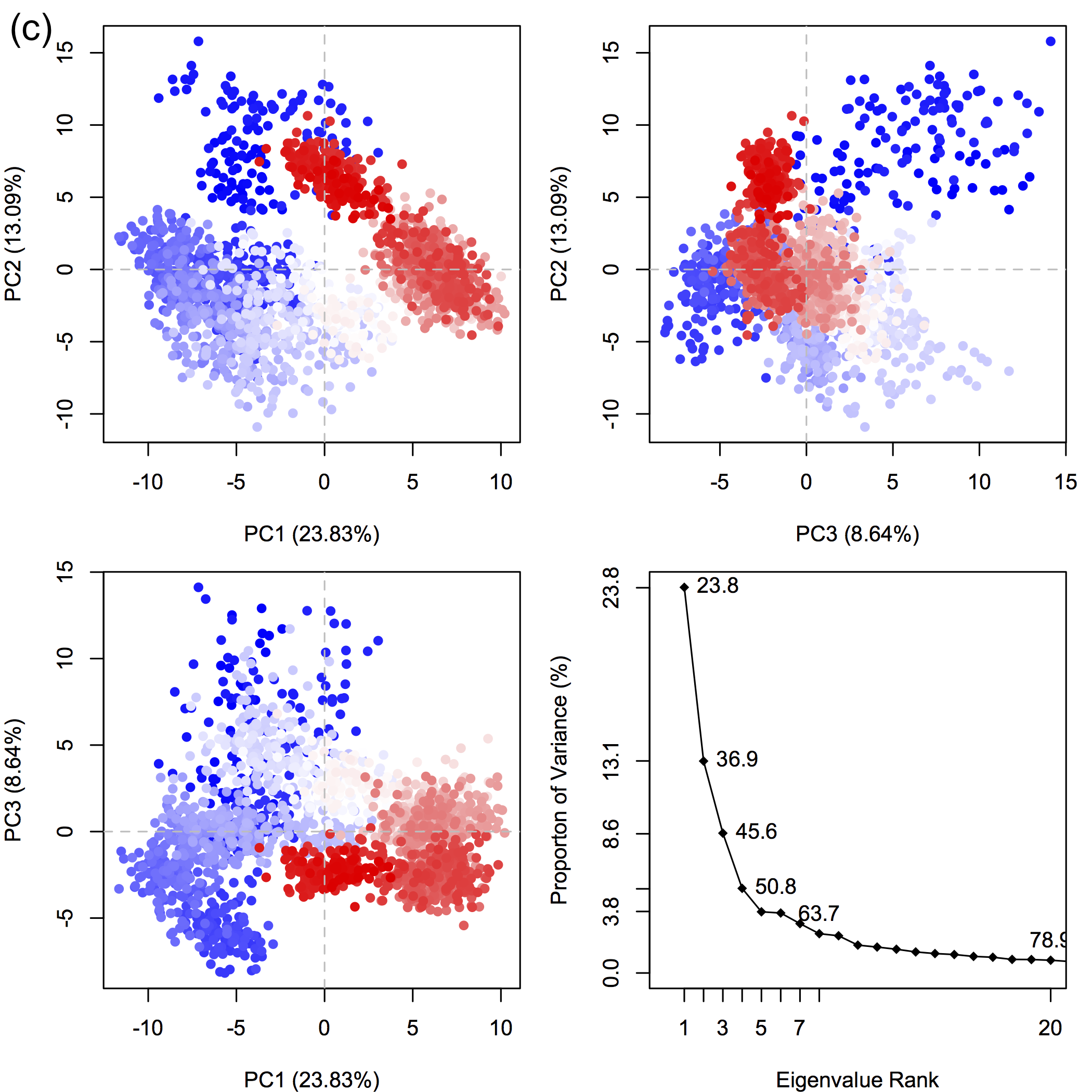

Supplement: S3 Fig — AgamOBP1 dimer in complex with 6-MH. PCA scatterplots. (DOCX) [file pone.0194724.s014.docx]

**S7 Fig. Secondary structure content (DSSP)**

**AgamOBP1-DEET multiligand complex**

**
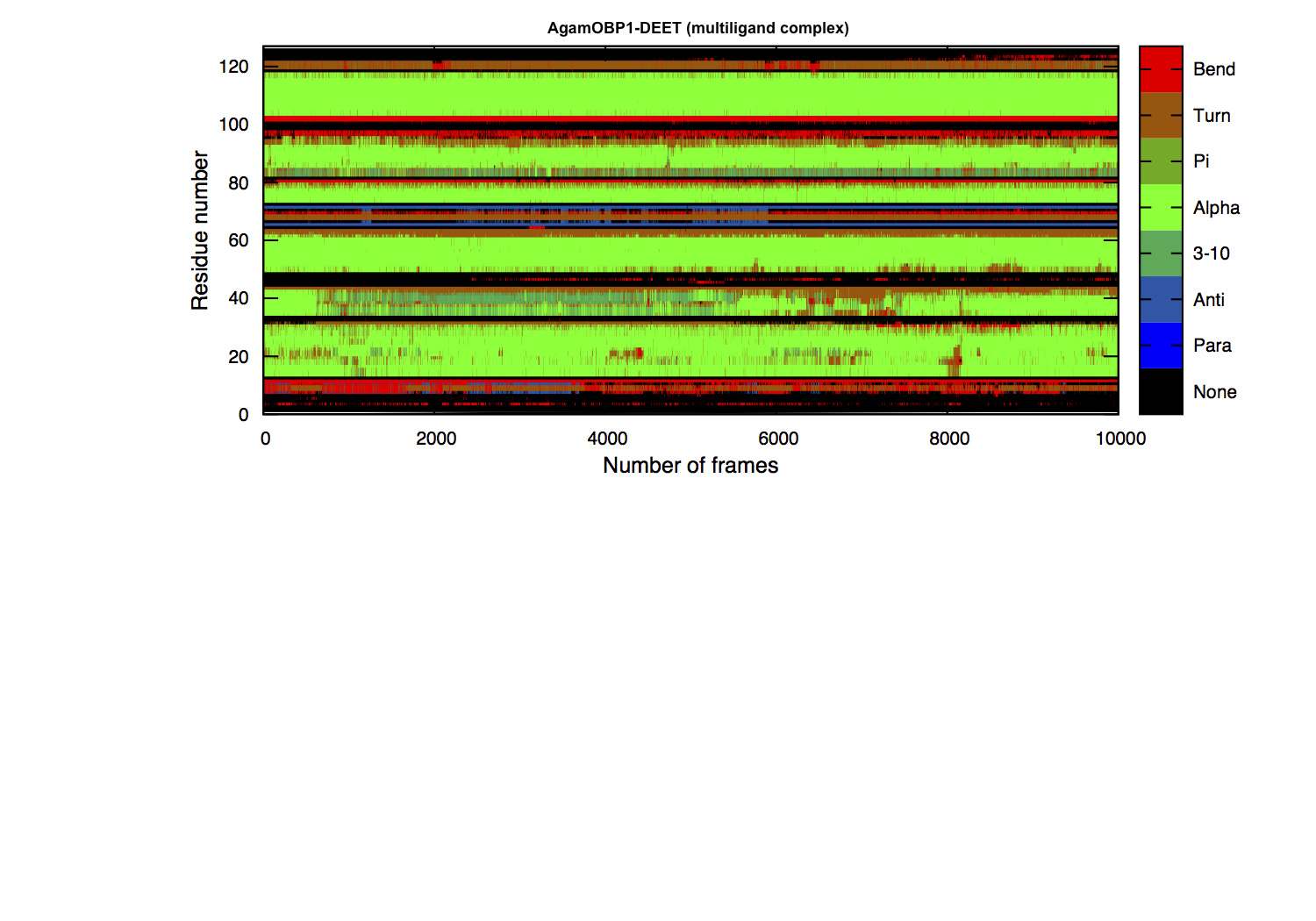
**

Supplement: S7 Fig — AgamOBP1-DEET multiligand complex. (DOCX) [file pone.0194724.s018.docx]

**S8 Fig. Secondary structure content (DSSP)**

**AgamOBP1-6MH multiligand complex**

**
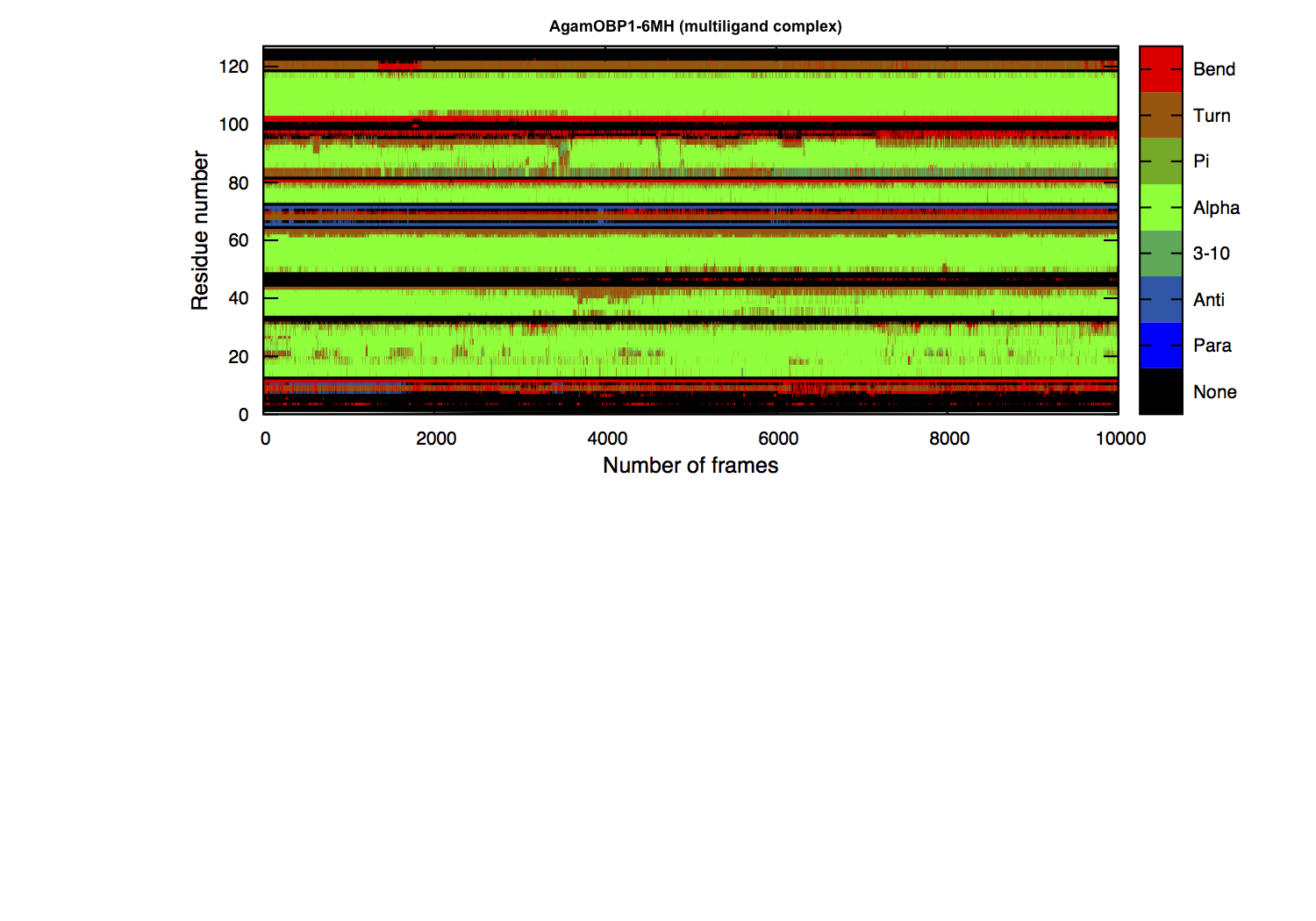
**

Supplement: S8 Fig — AgamOBP1-6MHmultiligand complex. (DOCX) [file pone.0194724.s019.docx]

**S10 Fig. AgamOBP1-DEET multiligand complex**

**PCA scatterplots**

**
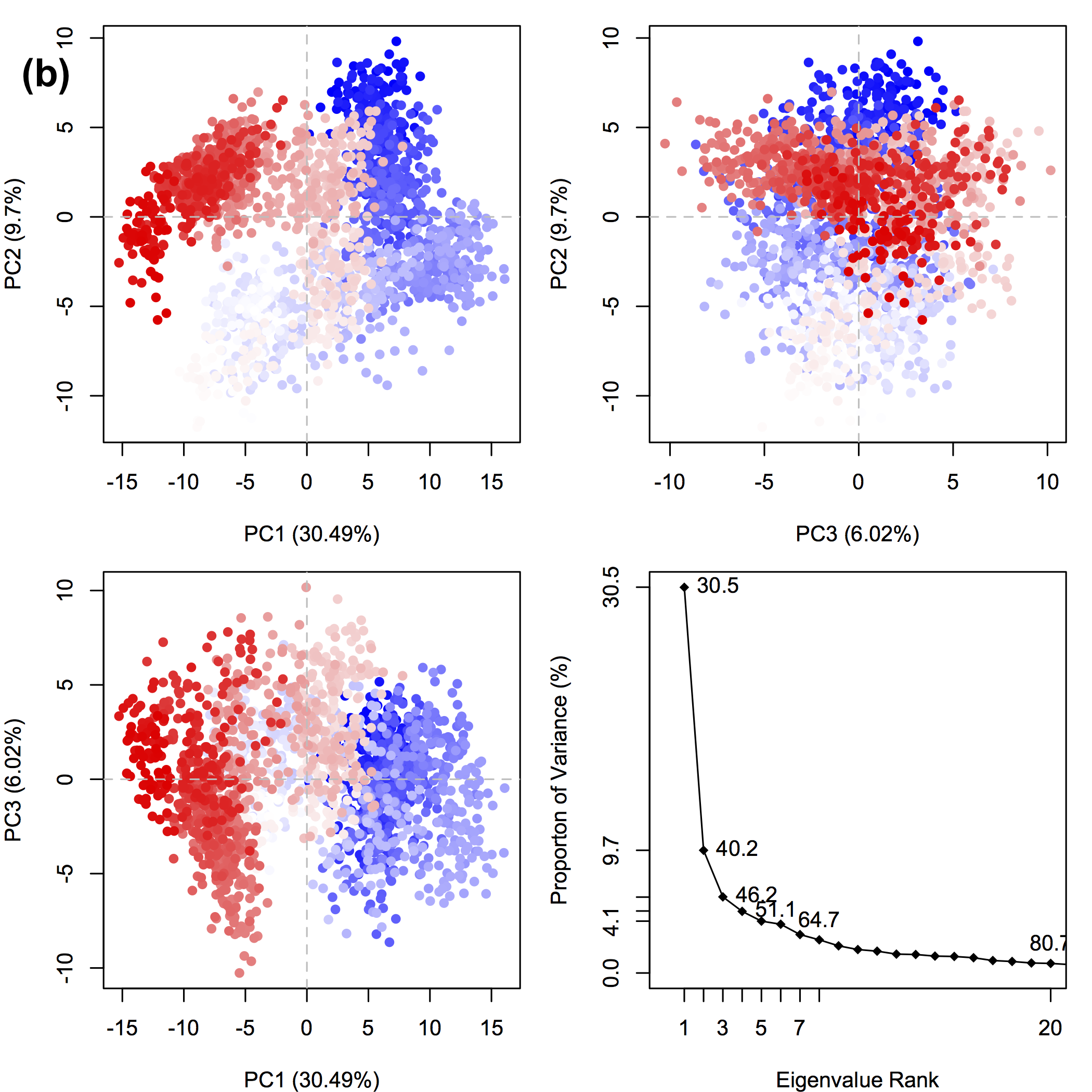
**

Supplement: S10 Fig — PCA scatterplots. (DOCX) [file pone.0194724.s021.docx]

**S11 Fig. AgamOBP1-6MH multiligand complex**

**PCA scatterplots**

**
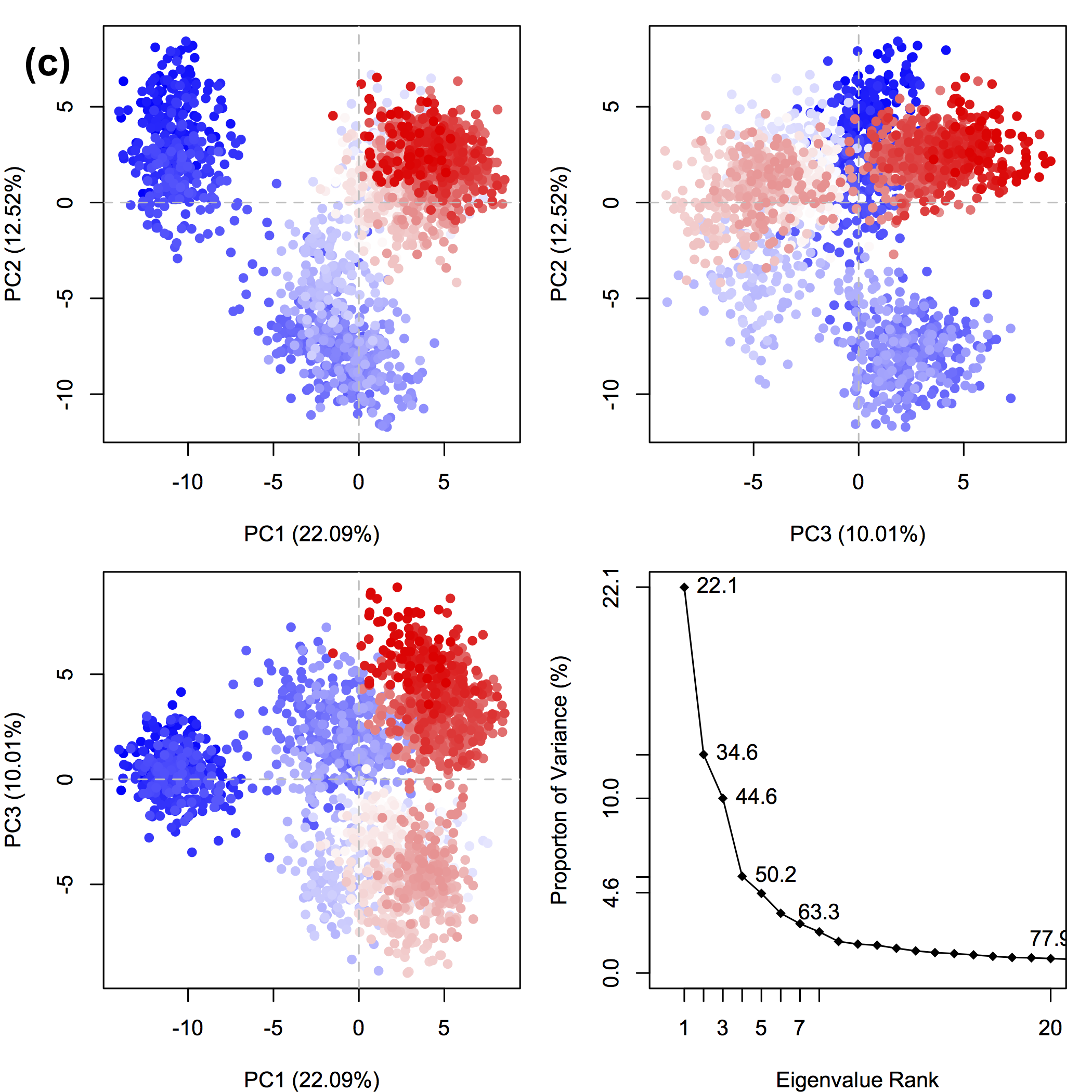
**

Supplement: S11 Fig — PCA scatterplots. (DOCX) [file pone.0194724.s022.docx]

**S14 Fig. CpHMD simulations.**

**Secondary structure content (DSSP) of AgamOBP1 apoprotein at pH 7 and pH 5**

**
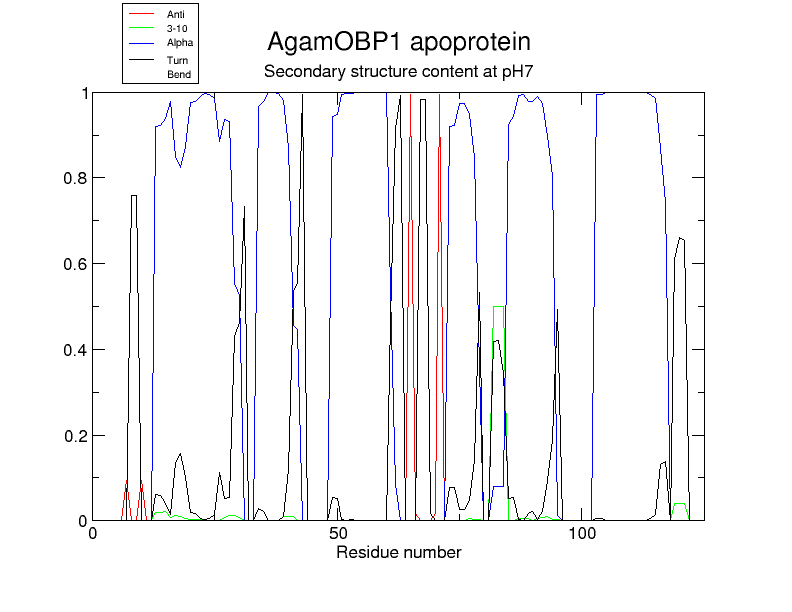

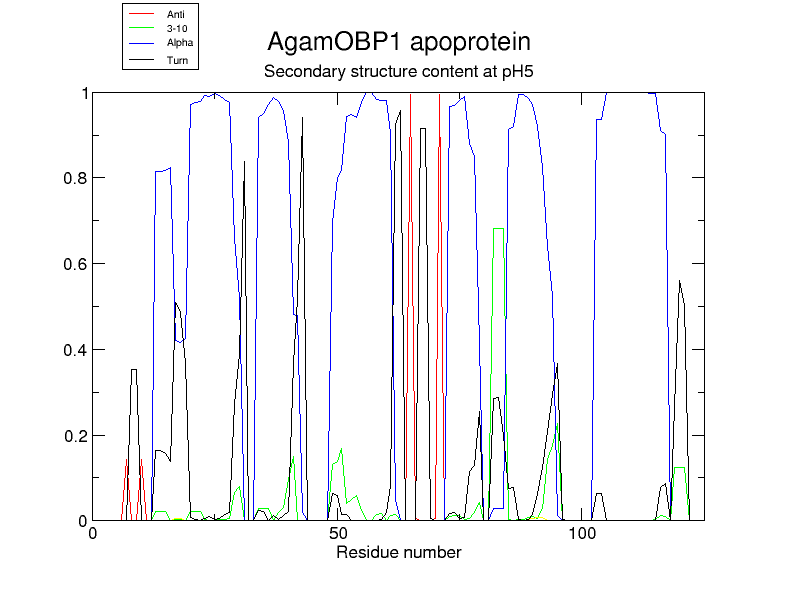
**

Supplement: S14 Fig — Secondary structure content (DSSP) of AgamOBP1 apoprotein at pH 7 and pH 5. (DOCX) [file pone.0194724.s025.docx]
