# Supplementary material for: New insights on repellent recognition by Anopheles gambiae odorant-binding protein 1
Source: PLoS One. 2018 Apr 3;13(4):e0194724. doi: 10.1371/journal.pone.0194724 (PMC5882127; doi:10.1371/journal.pone.0194724)
Supplement: S5 Fig — AgamOBP1 dimer in complex with 6-MH (subunit A). (DOCX) [file pone.0194724.s016.docx]

**S5 Fig. Secondary structure content (DSSP)**

**AgamOBP1 dimer in complex with 6-MH (subunit A)**


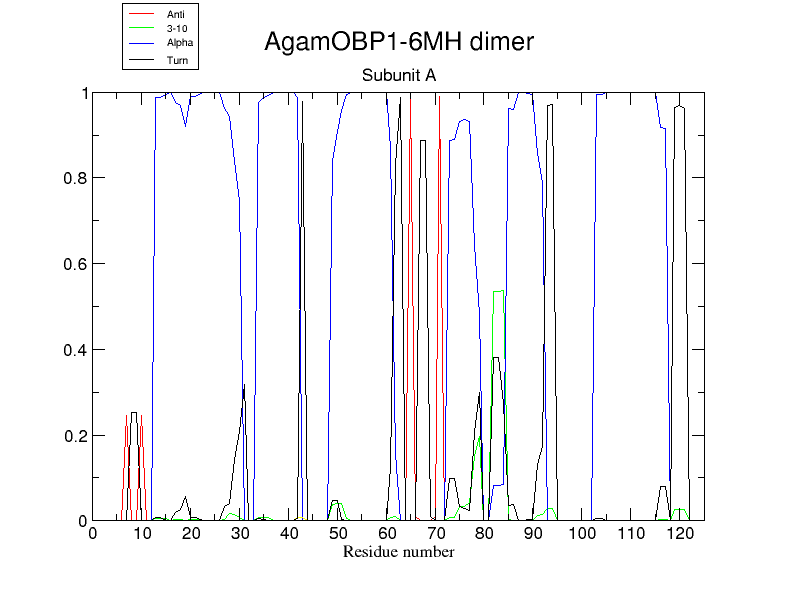

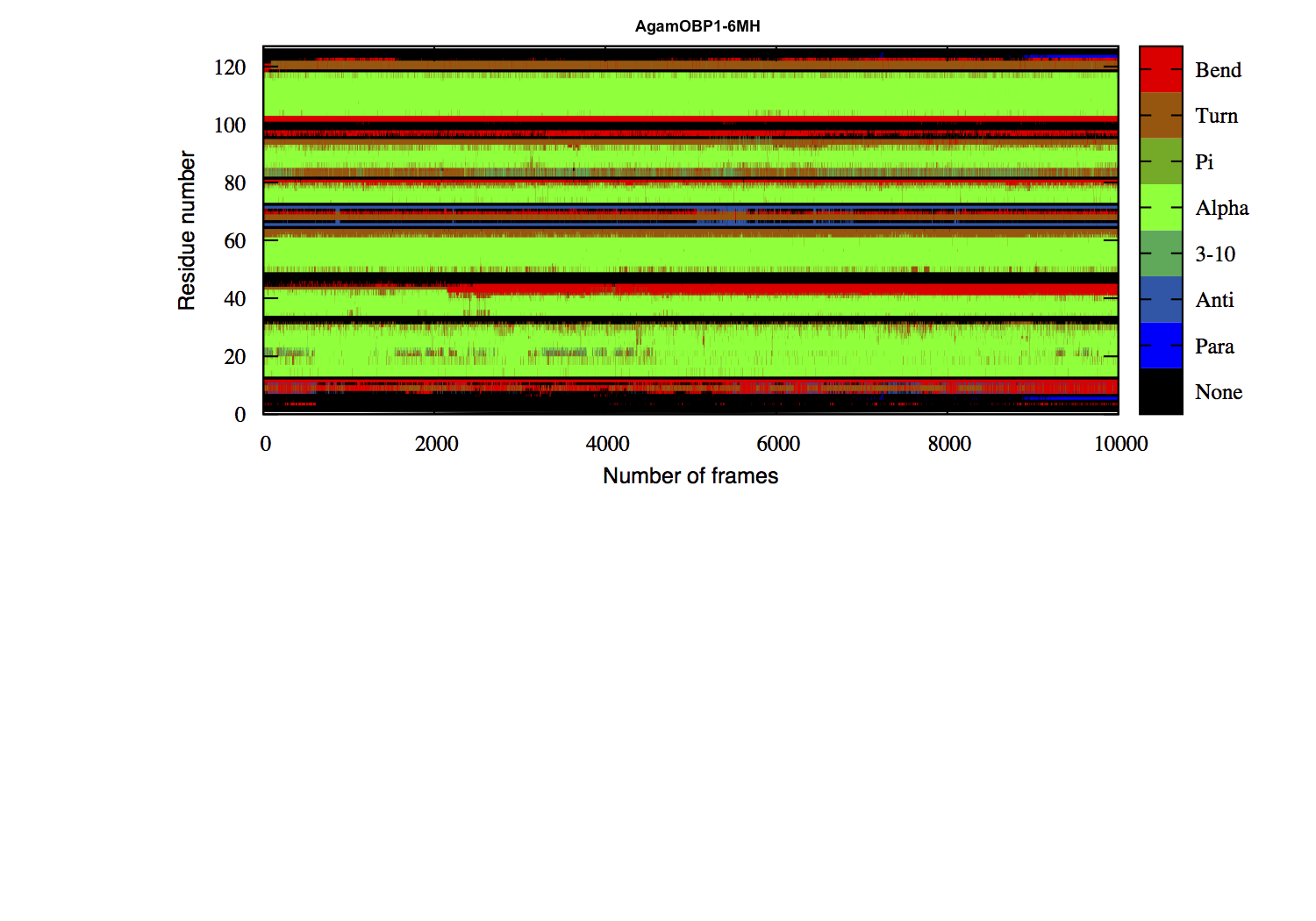
Two sets of MD simulations (Top: first simulation; bottom: repeat simulation)
