# Supplementary material for: New insights on repellent recognition by Anopheles gambiae odorant-binding protein 1
Source: PLoS One. 2018 Apr 3;13(4):e0194724. doi: 10.1371/journal.pone.0194724 (PMC5882127; doi:10.1371/journal.pone.0194724)
Supplement: S9 Fig — Principal Component 1: Comparison of most variable regions. (DOCX) [file pone.0194724.s020.docx]

**S9 Fig. AgamOBP1 multiligand complexes (DEET / 6-MH)**

**Principal Component 1: Comparison of most variable regions**

**
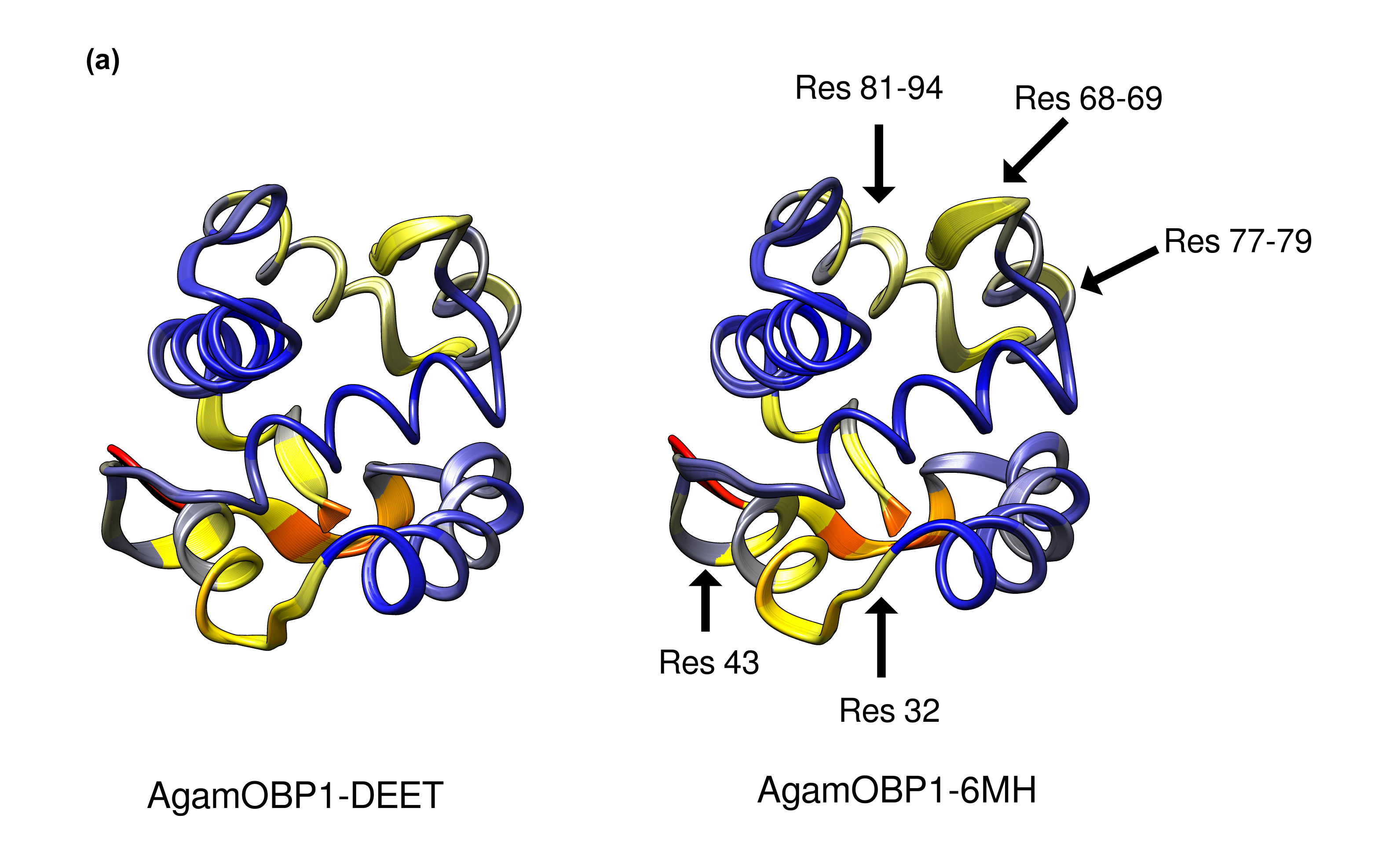
**
