# Supplementary material for: New insights on repellent recognition by Anopheles gambiae odorant-binding protein 1
Source: PLoS One. 2018 Apr 3;13(4):e0194724. doi: 10.1371/journal.pone.0194724 (PMC5882127; doi:10.1371/journal.pone.0194724)

**S12 Fig. CpHMD simulations**

**AgamOBP1 monomer. Time-series of RMSD of backbone atoms of AgamOBP1 from the starting structures over 20 ns of MD simulations**

| **pH** | **Ligand** | **Residues** | **RMSD_avg_ (nm)** | **σ (nm)** | **RMSD_min_ (nm)** | **RMSD_max_ (nm)** | **Time (ns)** |  |
| --- | --- | --- | --- | --- | --- | --- | --- | --- |
| 7 | None | 11-125 | 0.16 | 0.02 | 0.09 | 0.23 | 20 |  |
| 5 | None | 11-125 | 0.17 | 0.02 | 0.09 | 0.25 | 20 |  |
| 7 | Icaridin | 11-125 | 0.26 | 0.02 | 0.19 | 0.34 | 20 |  |
| 5 | Icaridin | 11-125 | 0.24 | 0.03 | 0.16 | 0.36 | 20 |  |
| 5 | Icaridin | 11-125 | 0.25 | 0.02 | 0.18 | 0.33 | 20 |  |
| 5 | Icaridin | 11-125 | 0.30 | 0.03 | 0.16 | 0.37 | 20 |  |
| avg = average rmsd value; σ = standard deviation. | | | | | | | | |


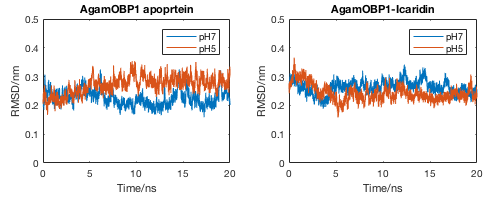

Supplement: S12 Fig — AgamOBP1 monomer. Time-series of RMSD of backbone atoms of AgamOBP1 from the starting structures over 20 ns of MD simulations. (DOCX) [file pone.0194724.s023.docx]
