# Supplementary material for: New insights on repellent recognition by Anopheles gambiae odorant-binding protein 1
Source: PLoS One. 2018 Apr 3;13(4):e0194724. doi: 10.1371/journal.pone.0194724 (PMC5882127; doi:10.1371/journal.pone.0194724)

**S13 Fig. CpHMD simulations**

**RMSF of backbone atoms of subunit A of the AgamOBP1 apoprotein and in complex with Icaridin at pH 7 and pH 5**


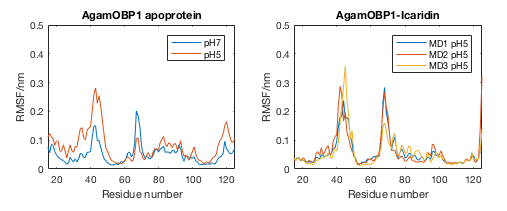

Supplement: S13 Fig — RMSF of backbone atoms of subunit A of the AgamOBP1 apoprotein and in complex with Icaridin at pH 7 and pH 5. (DOCX) [file pone.0194724.s024.docx]
